# A Machine-Learned Symbolic Committor for a Chemical Reaction: Isomerization in Retinal and *p*‑Coumaric Acid

**DOI:** 10.1021/acs.jctc.6c01496

**Published:** 2026-10-30

**Authors:** Kai Töpfer, Gianmarco Lazzeri, Vittoria Ossanna, Christopher J. I. Bläß, Florian Renner, Gianluca Lattanzi, Roberto Covino, Bettina G. Keller

**Affiliations:** † Department of Biology, Chemistry and Pharmacy, 9166 Freie Universität Berlin, 14195 Berlin, Germany; ‡ Institute of Computer Science, 9173 Goethe University Frankfurt, 60325 Frankfurt am Main, Germany; § 505684 Frankfurt Institute for Advanced Studies, 60438 Frankfurt am Main, Germany; ∥ Department of Cellular, Computational and Integrative Biology, 19034 University of Trento, 38123 Trento, Italy; ⊥ Department of Physics, University of Trento, 38123 Trento, Italy; ¶ Cluster of Excellence SubCellular Architecture of Life (SCALE), Goethe University Frankfurt, Frankfurt 60629, Germany

## Abstract

The thermal *cis*-*trans* isomerization of C=C double bonds in chromophores is a prototypical high-barrier reaction whose mechanism hinges on subtle out-of-plane bending motions. Combining machine-learning-guided path sampling (AIMMD) with symbolic regression, we learn an interpretable committor for two chemically distinct chromophores, *N*-retinylidene-lysine and *p*-coumaric acid anion, in vacuo from unbiased molecular dynamics trajectories generated by two-way shooting. Parametrizing the committor with a neural network and sampling it uniformly across the full range allows the model to resolve the reaction coordinate across the full transition region, not only at the isocommittor surface *p_B_* (**x**) = 0.5. A feature-attribution analysis identifies four proper dihedrals around the reactive bond as the informative coordinates, while the improper dihedrals at the sp^2^-hybridized carbon atoms prove unsuitable because reactant, transition, and product states share the same values. Symbolic regression then distills the committor into compact analytical expressions—to our knowledge the first committor both learned and distilled into closed form for a chemical reaction—and shows that a coupling of all four dihedrals is required to reproduce the S-shaped, stepwise pathway seen in the transition-path ensemble. This S-shape is absent from the minimum free-energy path: it arises from the dynamics of the short transition events combined with the mass asymmetry between heavy-atom and hydrogen-bearing dihedrals, and the larger asymmetry of *p*-coumaric acid yields the more pronounced S-shape this picture predicts. An interpretable, machine-learned committor, though a function of configuration alone, thus exposes dynamical features of the mechanism to which the free-energy surface is blind. The workflow requires no prior assumptions about the reaction coordinate and extends naturally to other high-barrier isomerizations and, combined with a reactive potential, opens a path toward bond-breaking chemical reactions.

## Graphical abstract

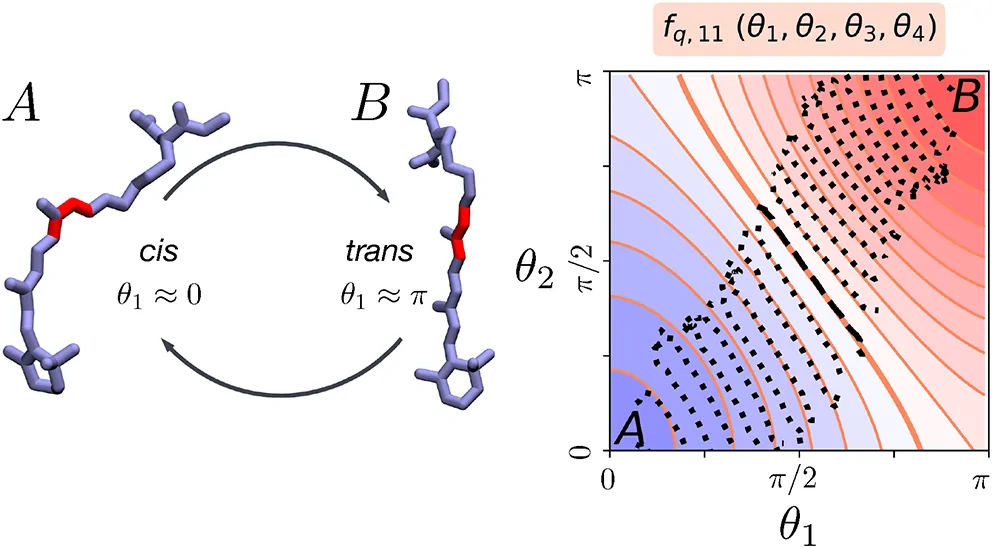

## Introduction

A rare event^1,2^ is a transition across an energy barrier that occurs infrequently because the barrier is much higher than the average thermal fluctuations. Chemical reactions are a prototypical but also a particularly challenging class of rare events.^3,4^ To rationalize their mechanism and to calculate reaction rates, one introduces reaction coordinates, i.e., functions of the conformational space that monotonically scale between the two energy basins on either side of the barrier and, ideally, report on the reaction dynamics. A faithful reaction coordinate turns a high-dimensional trajectory into a mechanistic picture and an accurate rate; a poor one yields neither. While it is usually possible to define a naive reaction coordinate by simply interpolating between reactant and product state, such a coordinate poorly captures the mechanism and often yields highly inaccurate rates. Finding optimal reaction coordinates is therefore a long-standing but still very active field of research.^5–7^ Minimum energy paths,^8–10^ traditionally used as models for the optimal reaction coordinate, identify the transition state (TS) as a saddle point on the potential energy surface but neglect entropic contributions and dynamical effects. The committor function^11–13^ instead, characterizes the actual dynamical flow of reactive trajectories: it is the probability that a trajectory initiated at a given configuration reaches the product before the reactant, and is widely regarded as the ideal reaction coordinate. The TS is the isocommittor surface at which trajectories are equally likely to reach reactant and product and the committor yields the mechanism at finite temperature together with accurate rate predictions in complex, high-dimensional systems.

Formally, the committor solves the backward Kolmogorov equation^14^ with absorbing boundary conditions on the two states, but obtaining it has long been considered intractable: it cannot be solved explicitly beyond a few degrees of freedom, and its direct estimation by shooting swarms of trajectories from every configuration is prohibitively expensive. Classical numerical approaches—direct solution of the committor equation on a grid, or its approximation on a discretized state space via Markov-state models and milestoning—are effective in low dimension but do not scale to the full configuration space of a molecule.^15–17^ Parametrizing the committor from molecular dynamics (MD) trajectories is correspondingly difficult,^3,18,19^ because the committor is a nonlinear function of the high-dimensional molecular state space.

Neural networks have been transformative in this context,^20–22^ as they can efficiently represent high-dimensional, nonlinear functions and make it possible to learn the committor directly from high-dimensional MD trajectories, without defining in advance a small set of predefined collective variables.^5^ Modern machine-learning strategies fall into three broad families. Variational, physics-informed networks minimize the backward-Kolmogorov functional^23–25^ and, coupled to committor-based enhanced sampling, now reach substantial barriers including genuine chemical reactions and nucleation.^13,26,27^ They are powerful, but require biasing the dynamics and evaluating coordinate gradients. A second family estimates the committor from ensembles of short trajectories,^19,28^ which is efficient but depends on those trajectories adequately covering the transition region. A third family infers the committor from the Bernoulli statistics of two-way shooting and couples it adaptively to transition-path sampling.^29–32^

In particular, some of us recently followed this last route developing “Artificial Intelligence for Molecular Mechanism Discovery” (AIMMD),^31,32^ a neural network that learns the committor of a rare event from short, dynamically unbiased trajectories generated by molecular simulations. A low-dimensional approximation of the committor is then obtained by symbolic regression, a genetic algorithm that searches both functional form and parameters to best fit the data. This provides a human-interpretable reaction mechanism expressed in terms of a few internal degrees of freedom.

To date, committors have been learned almost exclusively for biomolecular self-organization processes.^31–35^ These transitions are essentially diffusive: their barriers are comparatively broad, momenta relax quickly relative to the barrier crossing, and the dynamics is well described in the overdamped limit. In this regime the committor is a function of configuration alone and is the exact ideal reaction coordinate, so that a configurational, position-based model is both natural and sufficient. Most chemical reactions are different. Their barriers are steep and often both high and narrow, the transition events are correspondingly short, and the assumption of instantaneous momentum relaxation may break down during barrier crossing.^36^ In such inertial regimes the true committor is a function of the full phase-space point,^15,37,38^ and a committor that depends only on positions—the configurational committor obtained by averaging over the Maxwell–Boltzmann velocities—need not retain all the information that determines whether a configuration is reactive.^39^ Indeed, no purely configurational coordinate can render the dynamics free of recrossing,^40^ and in the gas-phase limit it is well established that a fully characterized potential energy surface does not by itself determine the mechanism, which is governed by the dynamics.^41–43^ It is therefore not obvious, a priori, whether a configurational committor can be both learned and predictive for high-barrier chemical reactions. The difficulty is compounded by the barrier itself: for high free-energy barriers the committor is essentially a step function that switches between 0 (reactant) and 1 (product), with only a narrow region around the TS taking intermediate values, so that a model capturing only the isocommittor surface *p*_*B*_(**x**) = 0.5 would appear accurate on most configurations while missing the mechanistic information that lives in that narrow interval.^44^ A further difficulty is interpretability. Even once learned, the committor is a high-dimensional, strongly nonlinear function; the neural network that represents it is accurate but opaque, and recovering from it a human-readable mechanism requires an additional step of analysis and distillation.^31,45^

As a stringent test case we study the thermal *cis*-*trans* isomerization around the C_13_=C_14_ double bond of retinal. Specifically, we consider the protonated retinal Schiff base covalently linked to a lysine residue, i.e., *N*-retinylidene-lysine, in vacuo (Figure 1a and Figure S1). It has a free-energy barrier^46^ of ∼100 kJ/mol in our force-field model. In addition, it has a complicated reaction coordinate. The intuitive reaction coordinate is the dihedral angle θ_1_ (Figure 1b) which cleanly separates reactant, transition, and product states but fails to accurately parametrize the probability flux across the TS.^46,47^ This is because near the TS, the reaction proceeds via out-of-plane bending motions of C_13_ and C_14_ (Figure 1c), which have to be included in order to obtain accurate rate estimates.^46^

**Figure 1:**
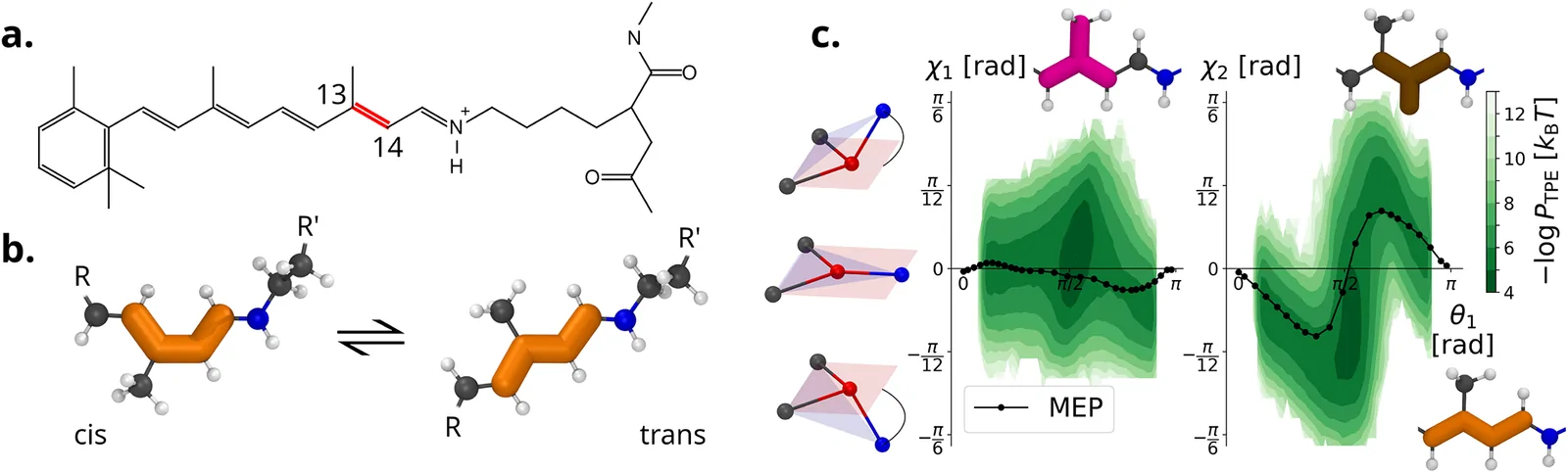
(a.) Lewis structure of retinal covalently linked to a lysine via a protonated Schiff base (*N*-retinylidene-lysine). The reaction center around the C_13_=C_14_ double-bond is shown in red with atom labeling. (b.) *Cis*-*trans* isomerization around the C_13_=C_14_ double-bond, with θ_1_ as naive reaction coordinate marked in orange. (c.) Minimum energy path (black) and logarithmic transition-path ensemble *F*_TPE_(***q***) = −ln *P*_TPE_(**q**) + *C* (green) of reactive trajectories projected into the space of the naive reaction coordinate θ_1_ and the improper dihedral angles, χ_1_ and χ_2_, centered at C_13_ and C_14_, respectively. See eqs 9 and 10.

The amplitudes of these motions are small and therefore difficult to detect in trajectory data. Accurately capturing them requires a model that is sufficiently expressive and trained on sufficiently informative data.

When described by the improper dihedrals χ_1_ and χ_2_, the out-of-plane bending appears as a flip-flop motion (Figure 1c), where the molecule forms a pyramidal distortion in one direction and, upon crossing the TS, reverses the distortion to the opposite side.^47^ The improper dihedrals χ_1_ and χ_2_ describe this motion. However, while the committor increases monotonically along reactive trajectories and thus provides a consistent measure of reaction progress, χ_1_ and χ_2_ do not. They assume values of χ_1_ = χ_2_ ≈ 0 in reactant, transition, and product states, and therefore cannot by themselves distinguish configurations with committor values of 0, 0.5, and 1. Consequently, they provide little information about reaction progress and are therefore not suitable for parametrizing the committor.

We complement retinal with a second, chemically distinct test case: the thermal *cis*–*trans* isomerization of *p*-coumaric acid anion (pCAA), the chromophore of the photoactive yellow protein^48^ and a prototypical photoreceptor switch, whose isomerization about the ethylenic double bond likewise crosses a high barrier. Retinal and pCAA differ markedly in size, substituent pattern, and conjugation, so that consistent conclusions across both point to general features of high-barrier isomerization rather than to idiosyncrasies of a single molecule.

Together, the two systems let us pose three questions: (i) Is the committor of a chemical reaction such as an isomerization learnable at all, given that for a barrier this high it is effectively a step function? (ii) Is the configurational committor—obtained with velocities averaged out—predictive and accurate for a system in which inertial effects may matter? (iii) Is the learned committor useful? That is, does it disclose something about the mechanism that we could not have obtained from the molecular structures or from the thermodynamic free-energy surface alone?

We present an in-depth analysis of how a neural-network model of the committor responds to these questions by modeling two chemically distinct *cis*-*trans* isomerizations, and show that learning the committor of such a high-barrier chemical reaction is both possible and practical. We show that a neural-network model, employed within an iterative sampling and learning framework, can both efficiently sample rare-event trajectories and yield a versatile, interpretable model of the committor that discloses the reaction mechanism of retinal isomerization beyond the transition state, and that the same approach carries over to the chemically distinct isomerization of pCAA.

## Theory

Consider a system with *N* atoms, where $x\in R^{3N}$ is a state in the 3*N*-dimensional positional space. We define two regions $A,B\subset R^{3N}$ with *A* representing the reactant state and *B* representing the product state. The system evolves according to a dynamical process for which the first hitting times τ_*A*_ and τ_*B*_ of the regions *A* and *B* are well-defined for all states outside these regions (**x** ∉ *A* ∪ *B*) (effectively integrating out the velocities).

The committor probability to the product state *B* for a process **x**_*t*_ starting in **x**_0_ at time *t* = 0 is

1 $$p_{B}\left(x\right)=P\left(\tau_{B}<\tau_{A}|x_{0}=x\right)$$

For any conformation within the reactant state *A*, the first hitting time satisfies $\tau_{A}\overset{\mathrm{def}}{=}0$ and hence *p*_*B*_(**x**) = 0. Likewise, for any within the product state *B*, $\tau_{B}\overset{\mathrm{def}}{=}0$ and hence *p*_*B*_(**x**) = 1. The complement of *p*_*B*_(**x**) is the committor function to the reactant state *p*_*A*_(**x**) = 1 – *p*_*B*_(**x**). Equation 1 represents the probability that a trajectory starting in **x** reaches *B* before *A*. The committor can be parametrized using the logistic function

2 $$\begin{array}{l} p_{B}\left(x\right)=\sigma \left(q\right)≔\frac{1}{1+e^{-q\left(x\right)}}\\ q_{B}\left(x\right)=\sigma^{-1}\left(p_{B}\right)≔\ln\frac{p_{B}\left(x\right)}{1-p_{B}\left(x\right)} \end{array}$$

where *q*_*B*_(**x**) is the logit of the committor probability to the product state *B*, or in short: the logit committor. The logit representation has been used previously to parametrize committor models and to resolve their behavior near the reactant and product basins, where the committor itself saturates.^28,31,49^ The transformation σ­(*q*_*B*_) and its inverse σ^–1^(*p*_*B*_) ensure that one can readily switch between the committor and the logit committor.

Evolving a single trajectory initiated at **x** is often referred as “(one-way) shooting”,^3^ and **x** as the “shooting point”. Initializing two trajectories from **x**, time-reversing one of the two due to microscopic time reversibility, and joining them together in a single reactive trajectory constrained to the shooting point is called “two-way shooting”. A one-way shooting result can be modeled as a Bernoulli trial with the two possible outcomes “*B* is reached before *A*” and “*A* is reached before *B*”, occurring with probabilities *p*_*B*_(**x**) and 1 – *p*_*B*_(**x**), respectively. The committor can therefore be inferred from trajectory outcomes using a maximum-likelihood approach.^29^

We represent the logit committor by a parametric model *q*_*B*_(**x**|**w**) with parameters **w**, which induces a parametric model of the committor *p*_*B*_(**x**|**w**) via eq 2. The training data consist of 2*k* trajectory outcomes δ_*ij*_ obtained from two-way shooting initialized from shooting points **x**_*i*_, where both forward and backward trajectories are treated as independent forward trajectories: *j* = 1, 2. Since the trajectories are generally initiated from *k* different configurations, each trial is characterized by its specific committor probability *p*_*B*_(**x**_*i*_|**w**), and we have two trials per shooting point initialized with opposite starting velocities. The negative log-likelihood of the data is therefore not binomial but given by the product of the Bernoulli probabilities of the individual outcomes

l(w|S)≔−ln⁡P(S|w)=−ln⁡∏i=1k∏j=12pB(xi|w)δij·(1−pB(xi|w))1−δij=∑i=1k∑j=12[δij⁡ln(1+e−q(xi|w))+(1−δij)ln(1+e+q(xi|w))]=∑i=1k[ri,B⁡ln(1+e−q(xi|w))+ri,A⁡ln(1+e+q(xi|w))] 3

where δ_*ij*_ is a binary outcome variable with δ_*ij*_ = 1 if the trajectory reaches *B* before *A*, and δ_*ij*_ = 0 otherwise. We substituted *p*_*B*_(**x**_*i*_|**w**) with eq 2 (see Supporting Information). The last line converts the binary trajectory outcome variables δ_*i*1_ and δ_*i*2_ into the shooting result **r**_*i*_ = (*r*_*i*,*A*_, *r*_*i*,*B*_) per shooting point, where **r**_*i*_ = (2, 0) if both trajectories end in *A*, **r**_*i*_ = (1, 1) if one trajectory ends in *A* and the other in *B*, and **r**_*i*_ = (0, 2) if both trajectories end in *B*. Equation 3 defines the loss function l­(**w**|*S*) and *S* = {(**x**_*i*_, **r**_*i*_)}_*i*=1_^*k*^ is the data set consisting of shooting points **x**_*i*_ and corresponding shooting result **r**_*i*_.

The data set *S* is generated by sampling transition paths and rare-event excursion paths using two-way shooting^3,50^ from selected shooting points **x**_*i*_. The probability that a sampled path is a transition path depends on the shooting point via^30^ *P*(TP|**x**) = 2*p*_*B*_(**x**)­(1 – *p*_*B*_(**x**)). This probability has a maximum at *p*_*B*_(**x**) = 0.5. By biasing the selection of shooting points according to *p*_*B*_(**x**), one can control the sampling rate of TPs.^29,30,32^

## *Cis*-*Trans* Isomerization in N-Retinylidene-Lysine

We begin by analyzing the *cis*-*trans* isomerizaton of N-retinylidene-lysine (Figure 1a,b), before turning to *p*-coumaric acid anion later in the text.

### AIMMD

AIMMD is based on an iterative sample and learning cycle (Figure 2a). This stage serves two purposes: (i) it generates a path ensemble consisting of transition paths and rare-event excursion paths, and (ii) it provides a reference neural-network representation of *q*_*B*_(**x**|**w**). The data set *S* = {(**x**_*i*_, **r**_*i*_)}_*i*=1_^*k*^ is directly derived from the path ensemble and remains fixed during the subsequent analysis (Figure 2b,c).

**Figure 2:**
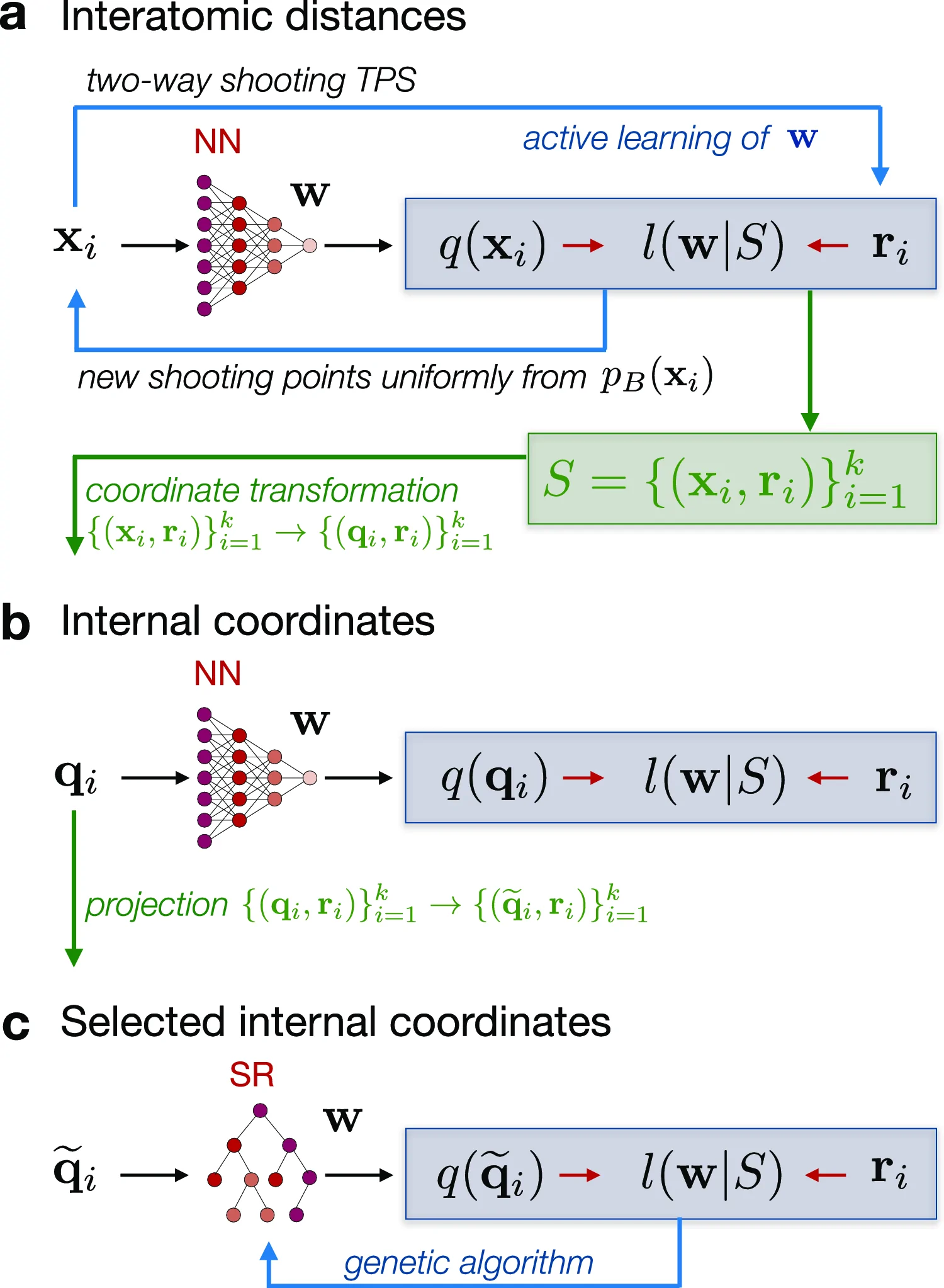
AIMMD. (a) Neural-network representation of the logit committor as a function of pairwise distances **x** and generation of the path ensemble {(**x**_*i*_, **r**_*i*_) }_*i*=1_^*k*^; (b) HIPR analysis using a neural-network representation of the logit committor as a function of internal coordinates **q**; (c) symbolic regression of the logit committor as a function of a reduced set of internal coordinates **q̃**_*i*_.

#### Generating the Path Ensemble

The iterative sample and learning cycle (Figure 2a) starts by training a NN of the logit committor *q*_*B*_(**x**|**w**), where **w** are the NN parameters, from one or several initial transition paths, obtained by, for instance, biased dynamics or interpolation. The NN subsequently guides the sampling of additional paths^31^ by selecting shooting point configurations **x**_*i*_ from previously generated reactive trajectories. From each selected configuration, new trajectories are launched using two-way shooting as in transition-path sampling (TPS).^3,51^ The selection probability is biased toward configurations close to the TS (*p*_*B*_ = 0.5), thereby sampling in high-energy configurations where accurate estimation of the logit committor is most critical. In contrast to standard TPS, shooting points can be drawn from all simulations, not only from previously accepted transitions, which accelerates exploration of the reactive region even further.^52^ Trajectories initiated from shooting points are terminated as soon as they reach the boundary of either region *A* or *B*, consistent with the relaxation time of the states, which renders them computationally inexpensive.^53^ Because no biasing forces are applied, the method reproduces the same rare excursions and transition events as an infinitely long unbiased simulation, albeit at a substantially higher rate.^32^ For the cis-trans isomerization of N-retinylidene-lysine, 9.1%out of 32,727 generated paths were transitions paths and constitute the transition-path ensemble (TPE). In principle, the transition yield could be further increased; however, this would come at the expense of stronger path correlations and consequently slower exploration of reactive phase space, resulting in a worse committor learning performance especially away from the TS.

The TPE shows the previously observed^46,47^ zigzag path in the (θ_1_, χ_1_) and (θ_1_, χ_2_) spaces (Figure 1c), confirming that TPS captures a similar reaction mechanism to that obtained with the path-collective-variable approach:^46^ torsion around the C_13_=C_14_ double bond with out-of-plane bending at the C_13_ and C_14_ carbons. This agreement allows us to use the TPE to further investigate the origin and consequences of out-of-plane bending.

#### Reference NN Representation of the Logit Committor

To ensure that neither *q*_*B*_(**x**|**w**) nor *S* is biased by prior assumptions about the reaction mechanism, the reference NN is trained on agnostic coordinates, namely the interatomic distances of the shooting-point conformations. Due to the high free-energy barrier in the retinal isomerization reaction, the committor approaches a step function,^14,54,55^ with values significantly different from 0 and 1 confined to a narrow region around the TS. This sharp separation makes accurate learning of the *p*_*B*_ = 0.5 isocommittor surface challenging. At the same time, complex reactive channels involve multiple nonlinear collective motions occurring sequentially,^47^ which span regions associated with both low and high committor values. Capturing only the TS region is therefore insufficient to describe the full reactive mechanism. To improve numerical stability and representation quality, we expressed the committor *p*_*B*_ in terms of the logit variable *q*_*B*_. In this representation, the reaction coordinate *q*(**x**) preserves variations in the input features more uniformly across configuration space **x**. This avoids a limitation of the committor *p*_*B*_, whose gradients vanish when approaching states *A* and *B*, making learning of the committor in these regions less effective. Due to the expressivity of *q*_*B*_(**x**), the NN that is trained during the iterative sampling and learning cycle in Figure 2a has a realistic chance to represent the reaction coordinate beyond the narrow TS region, provided that the training data are sufficiently diverse and extend away from it. In AIMMD, this goal is achieved by sampling approximately uniformly in the logit of the committor between two set interfaces, *q*_*B*,min_ = −20 and *q*_*B*,max_ = +20. Note that this reflects in a comparable number of shooting points being selected between *p*_*B*_ = 0.001 and *p*_*B*_ = 0.01 and between *p*_*B*_ = 0.01 and *p*_*B*_ = 0.1.

To validate this reference model, we estimated the logit committor for 438 starting structures **x**_*i*_ from an independent AIMMD run using 200 two-way shooting paths per **x**_*i*_ and compared the estimates to the NN prediction (Figure 3a). The comparison demonstrates that the model is highly accurate in the interval *q*_*B*_(**x**) ∈ [−4, 4] which corresponds to a committor probability interval of *p*_*B*_(**x**) ∈ [0.02, 0.98]. Estimates and predictions align particularly well in the central region of the plot near the TS. The slight deviations in the left and right regions of the plot appear as vertically stacked data points. This reflects finite-sampling effects in the committor estimator: while the NN model produces continuous predictions, the estimated committor values become effectively discretized for configurations strongly committed to either state, where only few shooting trajectories reach the opposite state.

**Figure 3:**
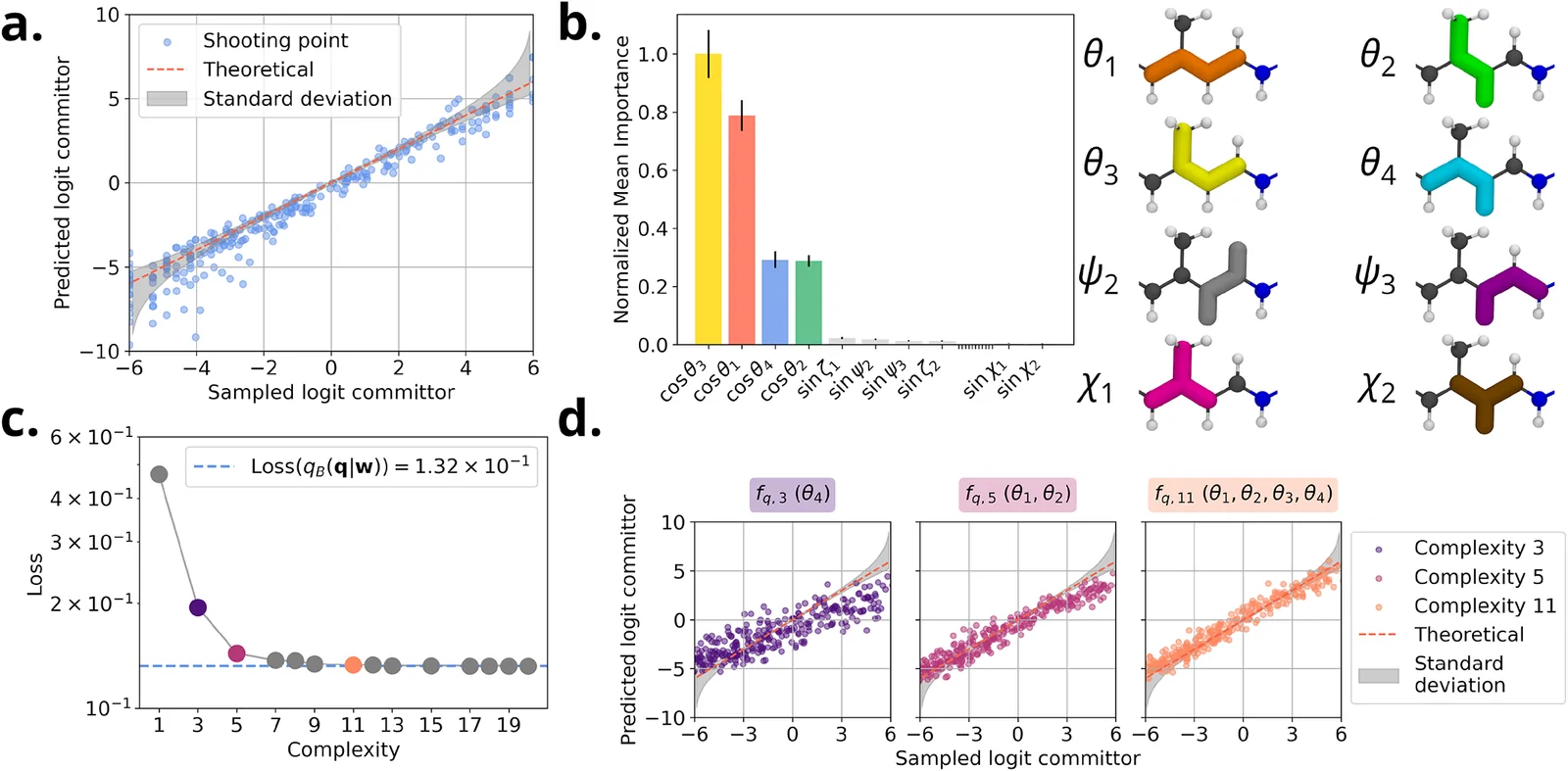
(a.) Correlation between sampled committor values and NN committor predictions of shooting point conformations (blue dots). (b.) Normalized absolute importance of the top 8 features from the HIPR analysis and the most important features of improper angles χ_1_ and χ_2_. Dihedral angles ζ_1_ and ζ_2_ involve atoms not shown in the atomic sketches, respectively. The black line indicates the standard deviation of the importance from the 10 individual runs. (c.) Comparison of best loss with increasing complexity of the committor function predictions from SR. (d.) Correlation between sampled committor values and committor predictions of shooting point conformers from the best SR functions of complexity (left to right) 3, 5, and 11, respectively.

Figure 3a is an extension of the classical committor histogram test.^56^ According to the latter, the committor distribution of shooting points with predicted committor values close to 0.5 should follow a narrow Gaussian distribution centered at 0.5, as is the case for our committor model (Figure S2a). Such a distribution indicates that the predicted *p*_*B*_ = 0.5 isocommittor closely coincides with the true transition-state ensemble. In contrast, an inaccurate reaction coordinate would produce a broad or even bimodal committor distribution, reflecting a mixture of reactant- and product-like configurations on the proposed transition-state surface.^56^ The narrow Gaussian distribution therefore provides strong evidence that the learned model accurately identifies the transition-state ensemble. Note, however, that the test reported in Figure 3a is more stringent, as it shows that our committor model is not only accurate around the TS, but over the entire range of the committor.

#### Identifying Relevant Internal Degrees of Freedom

Although the reference model approximates the logit committor accurately, it does not directly reveal the reaction center, the internal degrees of freedom that change during the reaction, or the sequence in which these structural changes occur. To extract the underlying reaction mechanism, we use the data set to train a second neural network (Figure 2b), but now represent the input in internal coordinates **q**, including distances, angles, proper and improper dihedral angles.

We use the same loss function as in the reference model (eq 3). Thus, the NN in Figure 2a,b differ only by a coordinate transformation of the input data **x** → **q**. Figure S3 shows that the new model *q*_*B*_(**q**|**w**) reproduces the sampled logit committor even more accurately than the reference model.

To evaluate the relevance of individual internal coordinates for the NN prediction, we then apply the holdback input randomization (HIPR) method,^57^ as in ref 31. In this analysis, the values of a specific internal coordinate *q*_*k*_ in the input data set are replaced by uniformly distributed random numbers within an interval [min*q*_*k*_, max*q*_*k*_] and the average loss of the modified input data set is calculated. Since the modified data set no longer reflects the learned relationship between **q** and *q*_*B*_(**q**|**w**), the loss is expected to increase relative to the loss obtained for the unmodified data. The magnitude of this increase indicates the importance of the corresponding coordinate for the NN representation of the logit committor. To reduce the impact of stochastic fluctuations, the procedure is repeated ten times for each internal coordinate and the loss is evaluated in each cycle. Crucially, HIPR identifies coordinates that are predictive of the logit committor. It does not assess whether a coordinate is geometrically meaningful or intuitive for describing the reaction pathway, nor does it minimize redundancy among the selected coordinates.

Only four internal degrees of freedom (cos θ_1_, cos θ_2_, cos θ_3_, cos θ_4_) exhibit a noticeable spike in importance in the HIPR analysis, whereas all other internal degrees of freedom have hardly any predictive power for the logit committor (Figure 3b). The four proper dihedral angles θ_1_ to θ_4_ (illustrated on the right of Figure 3b with matching color codes) correspond to the four possibilities to define the torsion around the C_13_=C_14_ double bond. Thus, HIPR correctly identifies the reaction center. The fact that neighboring torsion angles (proper dihedrals) contribute little to the model is consistent with previous analyses,^46^ which showed that the reaction center is very localized around the C_13_=C_14_-bond.

The improper dihedrals χ_1_ and χ_2_ at C_13_ and C_14_, respectively, exhibit low predictive relevance for the logit committor. This is expected, as the committor is not a monotonic function of χ_1_ and χ_2_, making them poor coordinates for parametrizing *q*_*B*_(**x**). In particular, reactant, transition, and product states all share χ_1_ = χ_2_ = 0, so this value provides little information about the progress of the reaction.

If the model *q*_*B*_(**q**|**w**) depends only on proper dihedral angles, does it still capture the out-of-plane bending at C_13_ and C_14_? In principle, the out-of-plane motion can be represented by a combination of proper dihedrals around the C_13_=C_14_ bond. The fact that the model relies on all four neighboring proper dihedrals suggests that the reaction coordinate is not trivially parametrized by a single torsion angle. However, HIPR does not resolve potential correlations between informative coordinates. Even though the high predictive quality of the model (Figure S3) indicates that the out-of-plane bending is likely included, we require an explicit representation of the logit committor as a function of internal coordinates to unambiguously identify the relevant degrees of freedom. Such a representation can be obtained via symbolic regression.

#### Explicit Representation of the Logit Committor

Symbolic regression (SR) is applied to express the logit committor function in a human-interpretable form using the most relevant internal degrees of freedom, determined by HIPR, as input.^31,45^ SR uses a genetic algorithm to both find the best representations in function and parameter space with respect to minimizing the same loss function (eq 3) as used for the NN models. The SR results are ordered by complexity (Figure 3c) which is measured as the total number of input variables, tunable parameter, binary operators (addition, subtraction, multiplication, division) and unary operators (e.g., exponential or logarithmic function). The SR algorithm is run 10 times to observe repeating patterns in the results. With increasing complexity, the estimated formulas by SR include higher number of input variables of which the least complex and best representation with respect to the lowest loss value are shown in eqs 4, 5, and 6 with one, two and all four of the variables cos θ_1–4_, respectively.

4a $$f_{q,3}\left(\theta_{4}\right)=a\cdot \cos\theta_{4}$$

4b $$f_{q,5}\left(\theta_{1},\theta_{2}\right)=b\cdot \left(\cos\theta_{1}+\cos\theta_{2}\right)$$

$$f_{q,11}\left(\theta_{1},\theta_{2},\theta_{3},\theta_{4}\right)=\left(c\cdot \cos\theta_{1}+\cos\theta_{2}-\cos\theta_{3}\right)\cdot \left(\cos\theta_{4}+d\right)$$ 4c

The parameters are *a* = 6.22, *b* = −5.76, *c* = −1.51 and *d* = 4.63. Figure 3d tests how well these analytical models of the logit committor reproduce the sampled logit committor.

The model with the lowest complexity that is still valid, eq 4, corresponds to a scaled single input variable cos θ_4_. Input variable cos θ_3_ could be also used for a valid logit committor function but with worse accuracy according to the loss function.

Equation 5 is the first model with two input variables and corresponds to a scaled sum of cos θ_1_ and cos θ_2_. Some SR runs also suggest a scaled sum of cos θ_3_ and cos θ_4_ but with a 0.1% higher loss value. The pairing of the dihedral angles is noteworthy: θ_1_ and θ_3_ consist of heavy atoms and contain C_12_. Since θ_1_ and, to a lesser degree, θ_3_ represent the main chain movement, it is understandable that the model assigns the highest significance to these two dihedrals. θ_2_ and θ_4_ both contain the hydrogen substituent at C_14_ and have a lower significance. In the analytical models with two input variables, a high-significance dihedral is paired with a low-significance dihedral, such that all four substituents to the C_13_=C_14_ double bond are represented.

Equation 6 is a model which combines all four input variables. In contrast to eqs 4 and 5, it is a nonlinear function of the input variables. Its loss value is about 8% lower than eq 5. Even though this decrease in loss seems modest in Figure 3c, in the visual comparison between predicted and sampled logit committor, eq 6 is the only analytical model that reaches comparable accuracy to the reference NN model. SR shows that, even though a 2-dimensional model of the committor is qualitatively correct, a 4-dimensional nonlinear model is required to be quantitatively accurate.

In total, we have generated two NN models and three analytical models of the logit committor. Pairwise comparison of these models and the comparison to the sampled logit committor are presented in Figure S3.

### Reaction Mechanism

Figure 4a shows the same TPE as in Figure 1c but now projected into the space of the dihedrals θ_1_ and θ_2_. The conformations of the two dihedrals are shown as Newman projections (θ_1_ = orange, θ_2_ = green) for selected points along the reaction. Carbon atoms are marked with black filled circles. In this projection, the zigzag motion in Figure 1c is transformed into an S-curved path which predominantly proceeds via θ_1_ in regions close to the reactant and product states but via θ_2_ around the TS. This stepwise mechanism generates the out-of-plane bending at the carbon atoms, since θ_1_ moves from 0 to almost π/2 while θ_2_ largely stays fixed. At the TS both dihedrals assume an angle of π/2.

**Figure 4:**
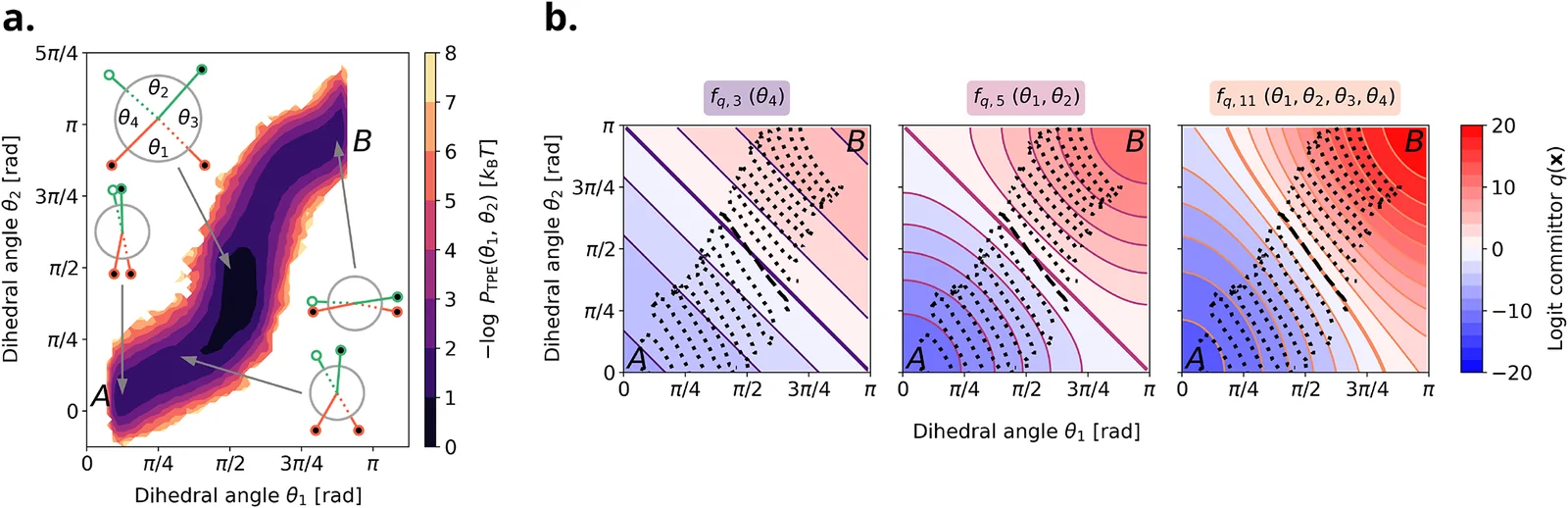
(a.) Logarithmic transition-path ensemble in the θ_1_, θ_2_ dihedral angle space (see eqs 10 and 9) with sketches of Newman projections of all 4 θ_*i*_ angles labeled in the projection of the TS (top left). (b.) Isocontour lines of the logit committor *q*(**x**) projected in θ_1_, θ_2_ dihedral angle space with θ_3_ = θ_4_ = π – (θ_1_ + θ_2_)/2 as ∑_*i*=1_^4^θ_*i*_ = 2π. The colormap and colored solid lines depict the isocontour lines of the logit committor according to the SR results with complexity 3, 5 and 11, respectively. The black dashed and dotted lines are the isocontour lines of the average logit committor values predicted by the NN logit committor model based on internal coordinates from conformations within the respective (θ_1_, θ_2_) bins. All lines depict a logit committor difference of Δ*q*_*B*_ = ±2 and the bold colored lines and black dashed lines indicate the logit committor of *q*_*B*_ = 0.

The NN model based on internal coordinates (Figure 2b) captures this S-curved reaction path, as demonstrated by the isocontours of the (average) logit committor in Figure 4b (black dots). Thus, despite the high reaction barrier, AIMMD successfully models details of the reaction mechanism beyond the TS ensemble at *p*_*B*_(**x**) = 0.5.

Figure 4b also shows how accurately the analytical models (eqs 4, 5, 6) reproduce the underlying (average) NN model predictions. While *f*_*q*,5_(θ_1_, θ_2_) (eq 5) is already a function of the (θ_1_, θ_2_) space, the other two models need to be slightly transformed to be represented in this space. For that, we use that the four dihedral angles sum up to a full circle (∑_*i*=1_^4^θ_*i*_ = 2π). This is evident from the Newman projection of the TS in Figure 4a. From the other Newman projections in Figure 4a, it is also evident that the opposing dihedrals approximately have the same value, i.e., θ_3_ ≈ θ_4_. Assuming that they have *exactly* the same value, we can assign their value in *f*_*q*,11_(θ_1_, θ_2_, θ_3_, θ_4_) as

5 $$\theta_{3}=\theta_{4}=\pi -\left(\theta_{1}+\theta_{2}\right)/2$$

The same relation allows us to convert any point in the (θ_1_, θ_2_)-space into a value of θ_4_ and insert it into *f*_*q*,3_(θ_4_) (eq 4). The isocontour lines of the resulting functions are shown in Figure 4b.

In *f*_*q*,3_(θ_4_) and *f*_*q*,5_(θ_1_, θ_2_), the reaction mechanism, defined by the gradient vectors orthogonal to the isocontour lines, follows a straight line connecting the *cis*-state at (0, 0) and the *trans*-state at (π, π). In doing so, these two analytical models fail to capture the S-shaped reaction pathway of the NN model. Even though the TS ensemble at *p*_*B*_(**x**) = 0.5 (i.e., *f*_*q*,3_(θ_4_) = *f*_*q*,5_(θ_1_, θ_2_) = 0) is well-aligned with the NN model predictions, the analytical approximations *f*_*q*,3_(θ_4_) and *f*_*q*,5_(θ_1_, θ_2_) smooth out the crucial feature that the reaction proceeds via a stepwise mechanism. *f*_*q*,5_(θ_1_, θ_2_) improves on *f*_*q*,3_(θ_4_) by capturing that the state boundaries of reactant and product states are approximately circular.

Only the model that uses all four dihedral angles identified by the HIPR analysis, *f*_*q*,11_(θ_1_, θ_2_, θ_3_, θ_4_), accurately captures the S-curvature of the reaction pathways. In this analytical form, the cosine terms are coupled multiplicatively (eq 6). This nonlinear coupling induces isocontour lines with an asymmetric curvature relative to the diagonal of the (θ_1_, θ_2_)-space. These isocontour lines perfectly align with the NN model, showing that the NN model can in fact be represented by a low-dimensional analytical expression.

### Origin of the Reaction Mechanism

We have established that the reaction follows an S-shaped pathway with a stepwise mechanism, associated with out-of-plane bending of the carbon atoms. The origin of this mechanism, however, remains unclear. This is particularly puzzling because the free-energy surface in the (θ_1_, θ_2_)-space does not exhibit an S-shaped profile (Figure 5a). Instead, the minimum free-energy path follows a straight line between reactant and product states. This contrasts with the TPE and suggests that the S-shaped reaction pathway does not arise from the underlying potential energy surface, but emerges from the dynamics.

**Figure 5:**
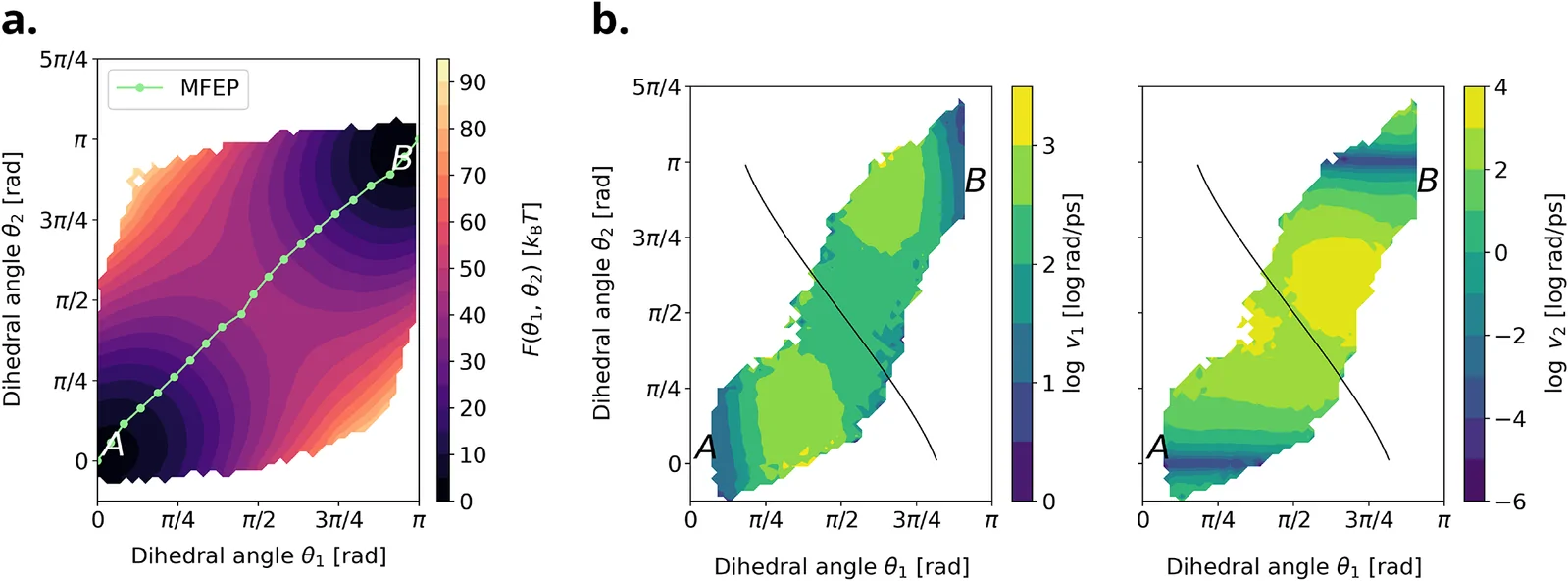
(a.) FES from a series of umbrella simulations and the minimum free-energy path (MFEP, dotted light green line) from NEB on the FES between (*A*) *cis* and (*B*) *trans* configuration. (b.) TPE average dihedral angle velocities (left) *v*_1_ = ⟨θ̇_1_⟩_TPE_ and (right) *v*_2_ = ⟨θ̇_2_⟩_TPE_ in frames with the respective (θ_1_, θ_2_) configurations. The solid black line marks the zero logit committor estimated by eq 6. Note the different color scale boundaries.

Figure 5b shows the average dihedral angle velocities *v*_1_ = ⟨θ̇_1_⟩_TPE_ and *v*_2_ = ⟨θ̇_2_⟩_TPE_ averaged over the TPE. The graphs highlight that, although both angles geometrically describe the same torsion around the C_13_=C_14_ double bond, they are not equivalent from the viewpoint of the reaction mechanism. First, the reaction mechanism is asymmetric at the configurational level: the system evolves predominantly along θ_1_ → θ_2_ → θ_1_, whereas the alternative sequence θ_2_ → θ_1_ → θ_2_ would correspond to a different reaction mechanism. Second, the velocity profiles of the two dihedral angles differ. ⟨θ̇_1_⟩_TPE_ reaches a maximum on either side of the TS, but θ_1_ is relatively slow at the TS itself. By contrast, θ_2_ has overall higher velocities and reaches its maximum velocity in the immediate vicinity of the TS.

We interpret these results as follows. First, the free-energy surface was reconstructed from umbrella-sampling. The velocities are therefore not selected by the rare-event kinetics and sample the equilibrium Maxwell–Boltzmann distribution. Second, the underlying two-way shooting simulations sample the equilibrium conditions, as initial velocities are drawn from the Maxwell–Boltzmann distribution and a thermostat is applied throughout. Thus, before conditioning on the outcome of a trajectory, the corresponding velocity distribution is likewise the equilibrium Maxwell–Boltzmann distribution. Third, transition-path sampling partitions this equilibrium path ensemble according to trajectory outcome. Reactive trajectories form the transition-path ensemble, whereas the remaining trajectories correspond to rare-event excursions. This splits the velocity distribution unevenly: particular combinations of configurations and velocities are preferentially associated with successful barrier crossing. Consequently, the velocity distribution conditioned on membership in the TPE is no longer Maxwell–Boltzmann distributed and can exhibit the position-dependent velocity profiles observed in Figure 5b.

Neither of the two subensembles is fully thermalized. Since the TPE is much smaller than the ensemble of nonreactive trajectories, deviations from the stationary distribution are more pronounced in the TPE. In this conditional ensemble, configurations near the transition state are overrepresented compared to equilibrium. The strong forces acting on the atoms near the reaction center rapidly convert kinetic into potential energy (and vice versa) within the reactive degrees of freedom. Because the transition paths are very short (0.128 ± 0.054 ps on average), this energy redistribution cannot equilibrate with the surrounding degrees of freedom or the thermostat. This inertial barrier-crossing dynamics generate the position-dependent velocity bias in the TPE.

Finally, the asymmetry between θ_1_ and θ_2_ arises from different effective masses associated with these degrees of freedom. While θ_1_ is defined in terms of four heavy atoms, θ_2_ contains the hydrogen substituent at C_14_. θ_1_ therefore has a higher effective mass than θ_2_. Visual inspection of individual transition paths reveals pronounced oscillations around the minimum energy path, indicating that the TPE should be interpreted in an average sense. With this caveat in mind, the velocity profiles suggest the following dynamical picture: the reaction is initiated by an excursion from the *cis*-state primarily along the heavier degree of freedom θ_1_, during which kinetic energy is converted into potential energy as θ_1_ approaches the transition region. The resulting strained, out-of-plane conformation is then relieved by a rapid transition of the hydrogen at C_14_, which generates the zigzag pattern in Figure 1c. This release of potential energy likely transfers kinetic energy back to θ_1_, facilitating barrier crossing and thereby completing the transition to the *trans* state.

## *Cis*-*Trans* Isomerization in *p*-Coumaric Acid Anion

To validate our findings and to add a second example, we model the thermal *cis*–*trans* isomerization around the C_2_=C_3_ double bond in *p*-coumaric acid anion (pCAA) (Figure 6). Similar to retinal, pCAA is a chromophore which undergoes fast *cis*-*trans* isomerization after excitation by visible light, and slow thermal *cis*-*trans* isomerization. The thermal reaction exhibits all challenges discussed in the Introduction section. However, the masses of the substituents around the reactive double bond differ more strongly than in retinal. In pCAA, one side of the double bond is connected to a relatively heavy 4-hydroxyphenyl group, resulting in a large effective mass for torsional motion along the main chain. In addition, each alkene carbon carries a hydrogen atom, whereas in retinal one of these hydrogen atoms is replaced by a methyl group. Consequently, the effective mass associated with the H–C_2_=C_3_–H torsion is expected to be smaller than that of the corresponding torsion in retinal. According to our previous analysis, these larger differences in effective masses should be reflected in the structure of the TPE and, consequently, in the reaction mechanism.

**Figure 6:**
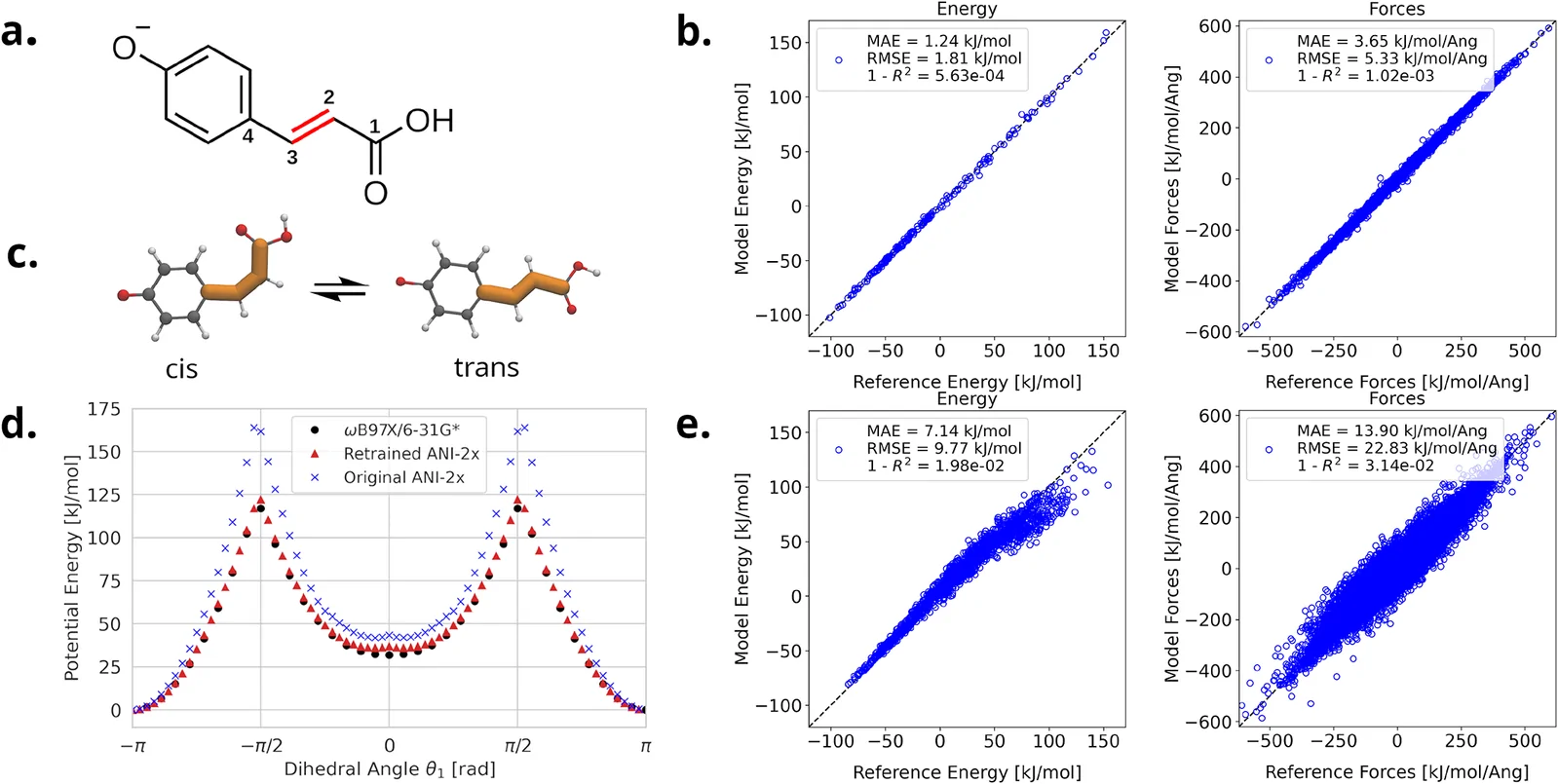
Machine-learned potential energy surface for *p*-coumaric acid anion. (a.) Lewis structure of pCAA with the carbons of the main dihedral numbered. (b.) Correlation plots of the reference and predicted energies (left) and forces (right) for the pCAA test set comprising 178 structures, obtained with the retrained ANI-2x model. (c.) pCAA in *cis* and *trans* conformation with the main-chain dihedral θ_1_ in orange (d.) Relaxed dihedral scans of pCAA. Black dots are the DFT method using the ωB97X/6–31G* functional and basis set. (e.) Correlation plots for energies (left) and forces (right) of 1500 random conformation samples of pCAA from the TPE (i.e., reactive trajectories only) generated with AIMMD.

### Machine-Learned Potential Energy Surface for pCAA

For retinal, we relied on a well-established empirical force field.^58^ No empirical force field of comparable accuracy is available for *p*-coumaric acid. To achieve the level of accuracy required for the present study, we therefore first constructed a machine-learned potential energy surface, representing a substantial additional methodological effort compared to the retinal study.

Specifically, we used the ANI-2x potential^59^ to sample configurations along the *cis*-*trans* reaction coordinate by metadynamics simulation with θ_1_ as the collective variable (CV), from which 1778 reference configurations were selected. Reference energies and forces were computed at the ωB97X/6–31G* level of theory and used to retrain the ANI-2x potential model. The transfer-learned potential reproduces both energies and atomic forces with low mean absolute and root-mean-square errors (Figure 6b), demonstrating that it accurately captures the reference potential energy surface within the configurational region represented in the training data.

In addition, we compared relaxed dihedral scans along θ_1_ obtained with DFT, the original ANI-2x potential, and the retrained ANI-2x potential. Note that the original ANI-2x potential is only trained on neutrally charged small organic molecules and is not supposed to predict accurately for the negatively charged pCAA. The retrained model, however, accurately reproduces the energy profile for configurations along the torsional coordinate governing the *cis*-*trans* isomerization (Figure 6d) with respect to the reference DFT energy profile. These results demonstrate that transfer learning successfully adapts ANI-2x to the conformational space and negatively charged electronic state relevant for the isomerization of pCAA.

As a stringent validation, we evaluated the transferability of the potential using random conformations extracted from the transition-path ensemble generated by AIMMD (Figure 6e). These structures were not part of the original training ensemble but were generated during reactive simulations with the transfer-learned potential itself. Although the deviations from the reference calculations are larger than for the training data, as expected, the energies and atomic forces remain well correlated with the DFT results. This demonstrates that the potential generalizes beyond its training configurations and remains sufficiently accurate in the transition region relevant for the reaction.

### Committor Model

We sampled the TPE of the thermal *cis*-*trans* isomerization in pCAA following the same AIMMD protocol as for retinal (Figure 2), yielding 7479 reactive paths sampling the TPE. We then constructed neural network and symbolic representations of the committor (Figure 7). The corresponding transition-state ensemble around *p*_*B*_(**x**) = 0.5, together with projection of the conventional PES saddle-point structure, is shown in Figure S2b of the Supporting Information.

**Figure 7:**
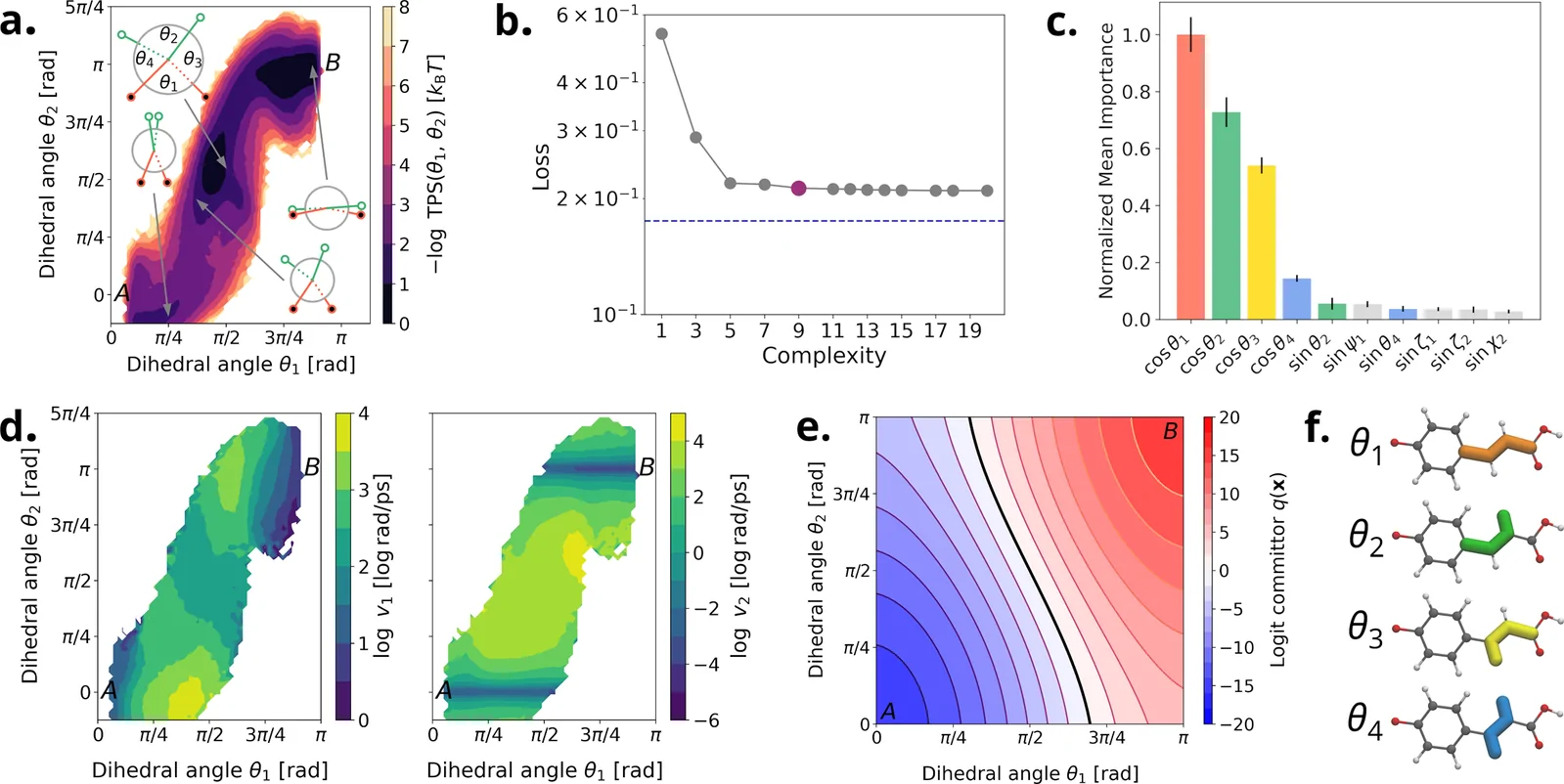
AIMMD analysis of *p*-coumaric acid anion. (a.) Logarithmic transition-path ensemble in the θ_1_, θ_2_ dihedral angle space. (b.) Comparison of best loss with increasing complexity of the committor function predictions from SR, with the dashed blue line at 1.75 × 10^–1^ being the minimal loss of the NN. (c.) Normalized absolute importance of the top 10 features from the HIPR analysis. The black line indicates the standard deviation of the importance from the 10 individual runs. (d.) TPE average dihedral angle velocities (left) *v*_1_ = ⟨θ̇_1_⟩_TPE_ and (right) *v*_2_ = ⟨θ̇_2_⟩_TPE_ in frames with the respective (θ_1_, θ_2_) configurations. Note the different color scale boundaries. (e.) Isocontour lines of the logit committor *q*(**x**) projected in θ_1_, θ_2_ dihedral angle space with the analytical form (cos θ_3_ – 2.52 cos θ_1_ – cos θ_4_)/0.30. (f.) Representation of dihedral angles θ_1_ to θ_4_, coloring matches the histogram in (c.).

The HIPR analysis of the neural-network committor identified the cosine of dihedral angles θ_1_ to θ_4_ as those internal degrees of freedom that have appreciable predictive power for the logit committor (Figure 7c,f). These dihedral angles θ_1_ to θ_4_ (illustrated in Figure 7f with matching color codes) correspond to the four possibilities to define the torsion around the C_2_=C_3_ double bond and define the reaction center. However, the predictive power decreases drastically across the four dihedral angles, and, in contrast to retinal, there is no gap between {θ_1_, θ_2_, θ_3_, θ_4_} and the other internal degrees of freedom, indicating that neighboring degrees of freedom might also be active in the TPE. This more broadly distributed predictive importance is consistent with the observation that the symbolic-regression loss remains above the neural-network loss at low and intermediate model complexities (Figure 7b). Reproducing the neural-network committor may therefore require additional coordinates, more complex functional relationships, or both.

The TPE in the two-dimensional space spanned by θ_1_, the main-chain dihedral angle, and θ_4_, the dihedral angle with the lowest effective mass, exhibits a pronounced S-shape (Figure 7a). The pronounced S-shape is consistent with our hypothesis that the effective-mass asymmetry influences the structure of the TPE. The corresponding velocity profiles provide further support for this interpretation (Figure 7d). The heavy coordinate θ_1_ accelerates before the transition state and decelerates as the transition state is approached, whereas the light coordinate θ_2_ undergoes its fastest motion near the transition state. Thus, the heavy and light coordinates traverse the transition region sequentially rather than synchronously, producing the stepwise, S-shaped reactive pathway also observed for retinal.

Figure 8a illustrates the reaction mechanism. The lower line focuses on the main chain movement from reactant state (*cis*) with θ_1_ ≈ 0° via the transition state with θ_1_ ≈ 90° to the product state (*trans*) with θ_1_ ≈ 180°. We additionally show two intermediate states left and right to the transition state, corresponding to the motion along θ_2_ in Figure 7. In these three states at the center, the main chain structure does not change, i.e., θ_1_ remains at 90°. The motion is in the C_2_ atom (orange dotted circle) which undergoes a flip-flop motion in its improper dihedral angle. The inset illustrates on this out-of-plane bending and shows that it can be interpreted as a (rapid) rotation of the hydrogen atom from left through planar to right. Focusing on the two hydrogen substituents at the C_2_=C_3_ double bond, one can see that this motion corresponds to a transition in the proper dihedral θ_2_: θ_2_ ≈ 0° at the first intermediate structure, ≈90° at the transition state, and ≈180° in the second intermediate state.

**Figure 8:**
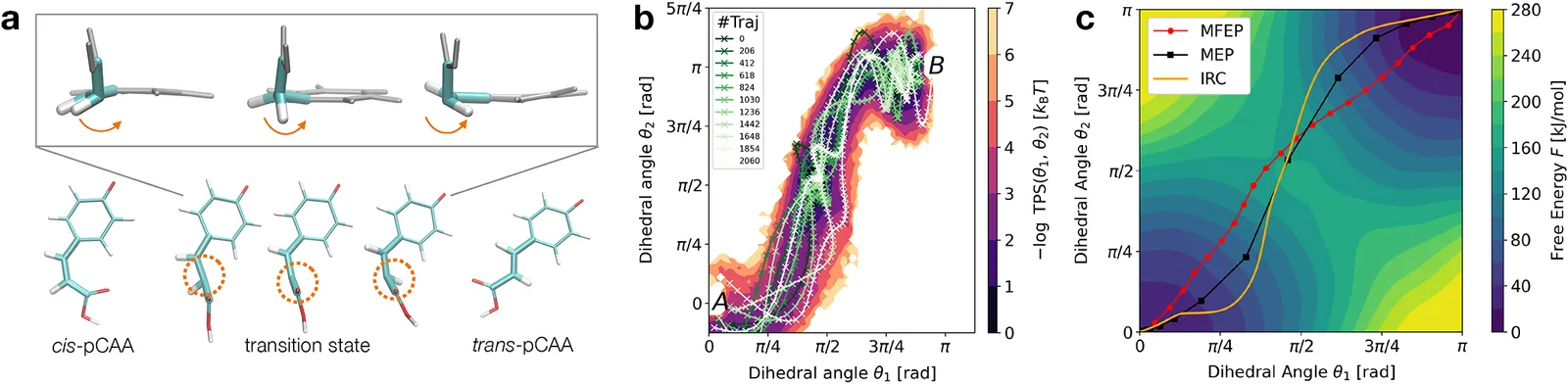
(a.) Reaction mechanism of the *cis*-*trans* isomerization in pCAA. The lower line focuses on the movement along the heavy main-chain torsion θ_1_. The inset illustrates the rapid movement of the hydrogen substituent at C_2_ along θ_2_. The view corresponds to a Newman projection along the C_2_=C_3_ double bond. (b.) Logarithmic transition-path ensemble projected onto the (θ_1_, θ_2_) dihedral-angle space, with representative reactive trajectories shown as individual paths. (c.) FES projected onto the same (θ_1_, θ_2_) space, together with the minimum free-energy path (MFEP), minimum energy path (MEP), and intrinsic reaction coordinate (IRC).

The close agreement between the intrinsic reaction coordinate (IRC) and the curvature of the transition-path ensemble (Figure 8b,c) further supports our interpretation that the S-shaped pathway arises from the mass asymmetry of the coupled torsional degrees of freedom, since the IRC is defined in mass-weighted coordinates.^60,61^ The TPE, however, contains additional dynamical information: individual reactive trajectories oscillate around this central path in the transition region. These oscillations provide a direct dynamical realization of the stable vibrational degrees of freedom assumed in Eyring–Polanyi transition-state theory^62,63^ and arise because thermal trajectories generally approach the saddle displaced from the minimum-energy reaction path.

## Conclusions

We set out to answer three questions about learning the committor of high-barrier chemical reactions. Studying two chemically distinct isomerization reactions—retinal and pCAA—enabled us to test whether our conclusions hold across systems rather than for a single molecule. First, the committor of such a reaction is learnable despite being effectively a step function: for both systems AIMMD resolves it not only at the transition-state ensemble but across the full reactive range. Second, the configurational committor—obtained with velocities averaged out—is itself learnable and accurate; its accuracy, however, does not imply that inertia is negligible, only that positions suffice to order configurations along the reaction. Third, the learned committor is useful in a strong sense: it discloses a stepwise, S-shaped mechanism that neither the molecular structures nor the free-energy surface reveals, and the comparison between retinal and pCAA turns the explanation we extract—inertial barrier crossing under mass asymmetry—from a single-system observation into a prediction that the second system bears out. We expand on each point below.

Using the *cis*-*trans* isomerization reactions of retinal and pCAA, we have demonstrated that AIMMD can efficiently sample a high-barrier chemical reaction and learn its committor accurately—not only at the transition-state ensemble *p*_*B*_(**x**) = 0.5, but well into the reactive region on either side. This is a nontrivial achievement: for barriers as high as those of these *cis*-*trans* isomerizations (∼100 kJ/mol for retinal^46^ and ∼125 kJ/mol for pCAA in our potential energy functions, see Figure 6d), the committor is effectively a step function, and a model that merely identifies the isocommittor surface at 0.5 would appear accurate on most test points while missing the mechanistic information that lives in the narrow interval around it. AIMMD avoids this failure mode by actively sampling configurations across the full committor range. Thorough TPE sampling and a smooth learning target together enable the model to resolve even the small-amplitude motions that shape the pathway—for both molecules.

The choice of coordinates through which the committor is expressed proved decisive. When viewed through the improper dihedrals at the sp^2^-hybridized carbon atoms, the small-amplitude motions appear as a flip–flop: reactant, transition, and product states all share χ_1_ = χ_2_ = 0, so these coordinates carry little information about reaction progress and are unsuitable for parametrizing the committor. The HIPR analysis revealed that the neural network instead relies on the four proper dihedrals around the C=C bond. In this representation, the pathway becomes monotonic from *cis* to *trans*, and what looks like a flip–flop in (χ_1_, χ_2_) is recognized as a fundamentally stepwise mechanism in the proper-dihedral space. The same reasoning holds for pCAA (Figure 8): the improper flip–flop of the hydrogen at C_2_ is recognized, in the proper dihedral (θ_1_, θ_2_), as an S-shaped monotonic transition from *cis*-configuration through the transition state to the *trans*-configuration.

Symbolic regression then distilled the neural-network representation into analytical expressions of the logit committor. Compared to the NN, these expressions are human-interpretable and computationally cheap, making them suitable as collective variables in biased simulations^39^ or for analytical rate estimates.^3,38^ They also extrapolate smoothly beyond the sampled region, where NN predictions become noisy—though the accuracy of such extrapolations, in the absence of sampling to check them against, remains an open question.

In accordance with our previous analysis,^46^ HIPR and symbolic regression reveal that the intuitive collective variable—a single dihedral angle around the reactive bond—is not a good reaction coordinate. For both systems, three to four proper dihedrals around the reactive bond are needed to accurately model the committor, although the separation between informative and uninformative coordinates is cleaner in retinal (Figure 3b) than in pCAA (Figure 7c), where predictive importance is more broadly distributed among neighboring torsions.

A cautionary finding emerged from this analysis: the loss is not a reliable indicator of whether an analytical model captures the mechanism. In retinal, the dominant reduction in loss occurs when moving from the one-coordinate model to the two-coordinate model, while the four-coordinate model is already far in the shallow regime of the complexity–loss curve. Nevertheless, this small reduction in loss between two- and four-coordinate models is mechanistically relevant. Only the four-coordinate model with nonlinear coupling terms between the coordinates reproduces the S-shaped reaction path; the two-coordinate model smoothes it into a straight line connecting reactant and product. In pCAA the difficulty appears from the other direction: the symbolic loss remains above the neural-network loss at low and intermediate complexities (Figure 7b), so no compact expression matches the network as closely as the four-coordinate form does for retinal. Chemically meaningful improvements are concentrated in the tail of the learning curve, which complicates overfitting control and model selection in symbolic regression for reaction-coordinate discovery.

For retinal, the committor can nonetheless be represented accurately by a remarkably simple symbolic expression of these four dihedral angles. This simplicity was not imposed a priori. Rather, the neural network was trained without restrictions on its functional form, and the symbolic model emerged solely from the data. For pCAA the same procedure yields a compact expression that captures the leading behavior of the committor but, consistent with the more distributed HIPR importance, does not reproduce the network as tightly (Figure 7b,e). Whether similarly compact symbolic representations are a general feature of chemical reaction mechanisms remains an open question.

To our knowledge, this work represents the first application of symbolic regression to a chemical reaction committor. Consequently, it is not yet possible to assess how broadly these findings generalize. While previous applications of symbolic regression to transition-state identification in soft-matter systems have likewise yielded compact low-dimensional models,^31^ additional studies on chemically diverse reactions will be required to determine whether this simplicity is a general characteristic of molecular reaction mechanisms or a particular feature of these isomerizations.

Perhaps the most striking observation of this work is that the free-energy landscape is, on its own, a misleading guide to the reaction mechanism. Deviations between thermodynamic pathways and dynamical reaction mechanisms are well-known from both chemical reaction dynamics^64^ and biomolecular conformational transitions,^65^ but they are often regarded as quantitative corrections rather than changes in the qualitative mechanism. Here, however, the discrepancy is sufficiently large to lead to qualitatively different mechanistic interpretations. The minimum free-energy path in (θ_heavy_, θ_light_) is a straight diagonal connecting *cis* and *trans*, suggesting a concerted torsion around the C=C bond. The transition-path ensemble tells a different story: reactive trajectories follow an S-shaped, stepwise path dominated by θ_heavy_ near the basins and by θ_light_ near the transition state. The discrepancy is not a failure of the free-energy calculation but a consequence of what the two objects represent. The FES integrates over all configurations at a given (θ_heavy_, θ_light_), including the overwhelming majority that belong to nonreactive fluctuations; the reactive density is masked by this nonreactive background. The TPE, by contrast, is conditioned on reactivity and therefore isolates the flow that actually produces the transition.

A second reason for the discrepancy is dynamical. The TPE is not a canonical ensemble: transition paths are too short for the strong forces near the reaction center to thermalize with the surrounding degrees of freedom. Combined with the mass asymmetry between the heavy-atom dihedral θ_heavy_ and the hydrogen-bearing θ_light_, this nonequilibrium regime produces the asymmetric θ_heavy_ → θ_light_ → θ_heavy_ sequence that underlies the mechanism. That this mechanism is driven by mass asymmetry, rather than by anything peculiar to retinal, is precisely what the second system was designed to test. Compared to retinal, the main-chain torsion θ_heavy_ has a larger effective mass because of the bulky 4-hydroxyphenyl group, while the substituent torsion θ_light_ has a lower effective mass, because the alkene substituents are only two hydrogens rather than a methyl-group and a hydrogen. Thus, pCAA exhibits a larger mass asymmetry than retinal. Our analysis therefore predicted a more pronounced S-shape for pCAA, and this is what its TPE shows (Figure 7a), with velocity profiles that again separate a heavy coordinate accelerating into the barrier from a light coordinate that is fastest at the barrier top (Figure 7d). The agreement across two molecules that share little beyond a reactive C=C bond indicates that the stepwise, S-shaped mechanism is a generic consequence of inertial barrier crossing under mass asymmetry rather than an idiosyncrasy of a single chromophore. The reaction mechanism is, in this sense, a dynamical phenomenon that neither the MEP nor the FES can reveal on its own. This echoes an insight from gas-phase reaction dynamics: even when the potential energy surface is well characterized, it does not uniquely determine the reaction mechanism, which is governed by the underlying dynamics.^41–43,64^

This picture answers the second question raised in the introduction. The configurational committor—velocities averaged out—proved indeed both learnable and accurate for both reactions, passing the committor-histogram test at *p*_*B*_ = 0.5 (Figure S2a) and reproducing sampled committor values across the full verifiable range (Figures 3a). Its accuracy should not, however, be read as evidence that inertia is irrelevant: away from the transition region, where *p*_*B*_(**x**) is close to zero or one, the outcome is already almost determined by the configuration and velocities can add little predictive information. Near the transition region, however, *p*_*B*_(**x**) ≈ 0.5, and the velocity along the reactive degrees of freedom can become highly informative, as expected for inertial barrier crossing. Indeed, Figures 5b and 7d show that the heavy and light torsional velocities evolve systematically as the system approaches and crosses the transition region. Given the high predictive power of the configurational committor, we expect the phase-space committor primarily to sharpen the dynamical description of the mechanism rather than to alter its qualitative configurational picture: the velocity along the reactive degrees of freedom should become increasingly predictive of commitment near the transition region, whereas the sequence of configurations identified from the configurational committor is expected to remain unchanged.

Consistent with this, the protocol incorporates equilibrium assumptions at several stages: shooting points are selected using the overdamped formulation of *P*(TP|**x**) and, more importantly, the logit committor is formulated solely in terms of positions, with velocities drawn from the Maxwell–Boltzmann distribution at shooting. Underdamped dynamics increases the region in which *P*(TP|**x**) is high.^36^ But since this probability is employed here solely as a sampling heuristic, it does not contribute to the loss function and therefore has no influence on the learned neural-network representation of the logit committor. By contrast, extending the framework to the phase-space committor^3,37,38^ is a natural next step, and one likely to matter more for high-barrier reactions than for the diffusive processes where AIMMD has previously been applied.

A second extension follows from AIMMD’s reliance on transition-path sampling, which naturally assumes a two-state description: multistate systems can be addressed by sampling transitions between multiple pairs of metastable states, for example within a core-set Markov model approach^16,17,66^ or through multiple-state TPS.^67^ Importantly, AIMMD can readily be applied to larger and more flexible molecules than retinal and to reactions which require an atomistically modeled environment, and—as the pCAA study demonstrates—to reactions for which no accurate empirical force field exists, at the cost of first constructing a machine-learned potential.

A further extension is to reactions that change molecular connectivity. Their main additional challenge lies in constructing an accurate potential energy surface that captures the associated electronic-structure changes, which can be particularly demanding for multiconfigurational transition regions. Once such a potential is available, however, transition-path sampling and committor learning are not fundamentally different from the present case: both describe barrier-crossing dynamics in a 3*N*-dimensional configurational space.

Taken together, our results show that learning the logit committor with neural networks, interpreting it with HIPR, and distilling it with symbolic regression offers a coherent route from unbiased molecular dynamics to a human-readable reaction coordinate. For both isomerizations, this route recovers the torsional mechanism and reveals its origin: the mechanism is shaped not by the energy landscape alone, but by the nonequilibrium, reactive dynamics that unfold on it—dynamics to which the free-energy surface is blind.

## Methods

### Retinal: Molecule and Force Field

The object of this investigation is the positively charged retinal-lysine Schiff base with the lysine saturated by an amide bond with acetic acid (N-terminus) and methylamine (C-terminus) shown in Figure 1b. The *cis*-*trans* isomerization reaction is defined by the change of dihedral angles θ_*i*_ around the C_13_=C_14_ bond. The potential energy surface of the molecule is provided by a modified AMBER99SB*-ILDN force field^68^ where the parameters of the retinal are adopted to GROMACS format^58,70^ from the work of Hayashi and co-workers.^71^ The initial molecular structure for retinal-lysine was obtained from experimentally measured crystal structure of channelrhodopsin 2.^72^

All MD simulations in AIMMD are carried out at 300 K in vacuum with GROMACS version 2018.8 (and 2019.6 for umbrella simulations)^73,74^ using the same settings as in ref 46. We simulated the system using the leapfrog stochastic dynamics integrator with a 2 fs time step and an inverse friction coefficient of 2 ps. The LINCS constraint algorithm was applied to constrain all hydrogen bonds.^75^

### Retinal: Minimum Energy Path

The minimum energy path (MEP) between the equilibrated *cis* and *trans* configuration of the retinal-lysine molecule is predicted by the climbing image nudged elastic band (ci-NEB) method implemented in the atomic simulation environment (ASE) version 3.25.0.^76,77^ The same set of AMBER force field parameters are used for the energy and force evaluation computed by its implementation in OpenMM version 8.2 and interfaced to ASE using the Narupa Python package.^78,79^

### Retinal: AIMMD

We used AIMMD for generating trajectory data while iteratively learning an initial NN representation of the logit committor. Then, we trained a final NN representation on the full training set. Finally, we used HIPR and symbolic regression to derive analytical formulas that serve as an approximation of the logit committor.

#### Data Production

The simulations focus on the *cis*-*trans* isomerization around the C_13_=C_14_ double bond of retinal, covalently coupled to a lysine residue. To characterize the system, we selected the dihedral angle θ_1_ ∈ (−π, π] defined by sp^2^–carbons 12, 13, 14, and 15 (see Figure 1). θ_1_ describes the isomerization process as expressed in the state definition function

6 $$\mathrm{SDF}\left(\theta_{1}\right)=\{\begin{array}{l} A,,\mathrm{if}\;|\theta_{1}|<\frac{\pi }{9},\left(cis\right)\\ B,,\mathrm{if}\;|\theta_{1}|>\pi -\frac{\pi }{9},\left(trans\right)\\ R,,otherwise,\left(reactive\right) \end{array}$$

AIMMD sampled the path ensemble that represents the retinal isomerization event from *A* via the TS at θ_1_ ∼ π/2 to *B*. (The opposite rotation was not considered). The algorithm requires an initial transition path (TP) to initialize the early simulations; this path does not need to represent a physically accurate trajectory. We generated the initial path from two-way shooting from a high energy configuration with θ_1_ ∼ π/2 and assumed to be at the TS. AIMMD generates paths by performing two-way shooting from previous trajectories. Simultaneously, it updates a NN as an intermediate step (Figure 2a), which in turn refines the shooting point selection based on the most recent NN model.

During the data production process, the NN model is a suboptimal approximation of reaction coordinate model *q*_*B*_(**x**). The model for the generation process (Figure 2a) employed a feedforward neural network (FFNN) with 3,160 input features corresponding to pairwise atom–atom distances. The model architecture is composed of three layers with PReLU activation functions. We employ ADAM^80^ for optimization with an initial learning rate equal to *l*_*r*_ = 10^–5^. At regular AIMMD steps, following each completed two-way shooting simulation, we retrain the networks from scratch using a loss function derived from the simulation outcomes (eq 3). The training set matches the number of simulated excursions and transitions, containing (**x**_*i*_, *r*_*A*,*i*_, *r*_*B*,*i*_) triplets. Here, **x**_*i*_ denotes the atom positions of the shooting point of the path, while *r*_*A*,*i*_ and *r*_*B*,*i*_ represent the counts of boundary crossings for states *A* and *B*, respectively. Subsequently, we minimize the loss function in eq 3 over 500 training epochs, resampling the training set without replacement in batches of size 4096.

We perform path sampling excluding equilibrium simulations. The sampling strategy and reweighting procedure ensure a uniform distribution in the *p*_*B*_(**x**) ≈ σ­(*q*_*B*_(**x**)) space, where *p*_*B*_ is an optimal reaction coordinate that defines the transitions between two states, between the manually defined interfaces *q*_*B*,min_ = −20 and *q*_*B*,max_ = +20. These interface values place the system sufficiently close to the states while avoiding direct proximity to the boundaries. These settings enable the system to sample the shooting point from a broader range of paths, rather than relying solely on the most recent path, which eventually ensures more extensive exploration of the reaction space and prevents stagnation near the states. This procedure may restrict exploration in directions orthogonal to the committor. Our simulations show that within the range of positive θ_1_ values, only one pathway exists, indicating rapid equilibration in that direction. Lazzeri et al. provide additional settings and detailed guidance on the methodology employed.^32^

The final data set consists of a total of 32,727 atomistic simulations representing shooting paths, with 9.1% classified as transitions and the remaining categorized as excursions around the state basin.

To project the transition-path ensemble density profile, we reweight the paths to ensure they populate the target ensemble in equilibrium proportions. The selection of shooting points follows the Boltzmann distribution along the isocommittor surfaces, as guaranteed by the theoretical framework underlying sampling in AIMMD.^52^ We assign a reweighting factor to the paths, proportional to the inverse of the number of crossings at the shooting interface. Here, we use an approach consistent with established methodologies,^81,82^ where the reweighting ensures proper Boltzmann-weighted sampling. After reweighting, AIMMD sampling becomes equivalent to transition interface sampling, with the interfaces aligned to the locations of the shooting points. This step precedes the path ensemble reweighting procedure described in detail by Lazzeri et al.^32^

To visualize the transition-path ensemble in a reduced coordinate space, we project all configurations along the reactive trajectories onto a set of collective variables (**q** = **q**(**x**)). The corresponding transition-path density is defined as

7 $$P_{\mathrm{TPE}}\left(q\right)={\left\langle \int_{0}^{\tau \left[x\right]}\delta \left(q-q\left(x\left(t\right)\right)\right)dt\right\rangle }_{\mathrm{TPE}}$$

where **x**(*t*) denotes a trajectory in the transition-path ensemble, τ­[**x**] is its duration, ⟨·⟩_TPE_ denotes an average over all transition paths. To estimate *P*_TPE_(**q**) from the TPE the paths must first be reweighted such that they contribute according to their equilibrium probability in the target ensemble following the procedure described in refs 30,81 . We then plot the effective free energy of the transition-path density

8 $$F_{\mathrm{TPE}}\left(q\right)=-\ln P_{\mathrm{TPE}}\left(q\right)+C$$

where *C* is an arbitrary additive constant.

#### NN Embedding of the Committor

In general, it is always possible to improve the model after the sampling campaign has ended.^33^ We retrained a custom FFNN implemented in PyTorch,^83^ comprising three fully connected layers. The network consists of an input layer, two hidden layers, each containing 512 nodes, and a one-node output layer. A dropout rate of 0.5 is applied after the input layer and the first hidden layer. After each layer, the network uses a PReLU activation function. We employed the ADAM optimizer with an initial learning rate of 10^–5^, training the model for 500 epochs with a batch size of 32. The input features were derived from the internal coordinate representation of the shooting points, including 80 bond distances, 144 angles, and 157 dihedral angles projected as sine and cosine components, resulting in a total of 538 features. The shooting results **r**_*i*_ = (*r*_*i*,*A*_, *r*_*i*,*B*_) were defined from the outcomes of the two-way shooting initiated at **x**_*i*_, capturing the frequency with which each path visits states *A* and *B*. We adopted the loss function used within the AIMMD framework and the same rewriting strategy. We split the data set based on the AIMMD production runs, with a ratio of 77:23 for training and testing, respectively. This division ensured that the correlation between the test and train data set was minimal.

We validated and assessed the accuracy of the committor learned by the NN using a dedicated validation set consisting of 438 shooting points excluded from prior steps. For each data point in the validation set, we performed a two-way shooting procedure, yielding 400 shooting outcomes, where *N*_*A*_ is the number of shooting outcomes that reach *A* before *B* and *N*_*B*_ is the number of outcomes that reach *B* before *A*. Of the 438 validation points, 175 yielded committor estimates of exactly (*p*_*B*_ = 0) or (*p*_*B*_ = 1); the remaining 263 points had finite logit-committor values and were used for the quantitative comparison with the neural-network predictions. The committor estimate *N*_*B*_/(*N*_*A*_ + *N*_*B*_) is compared to the neural-network prediction in Figure 3a/Figure S3 showing strong agreement between the shooting-based estimates and the model predictions.

#### Symbolic Regression

We then performed symbolic regression using the PySR framework.^84^ We injected the four most significant features identified through HIPR analysis as inputs for the symbolic regression, and we used the two-way shooting results as labels. To maintain methodological consistency, we applied the same AIMMD loss function to this optimization problem and preserved the same data set split into training and testing subsets. The symbolic regression framework permitted algebraic sum, multiplication, division, logarithm, and exponential functions, constraining the maximum complexity to 20 and maximum depth to 7. We performed 1000 iterations for each cycle. All other parameters remained at their default values.

To mitigate stochastic effects arising from system initialization, we executed ten independent symbolic regression runs using identical parameters. We assessed the trade-off between model complexity and performance through validation set evaluations, ensuring a robust analysis of the derived analytical expressions.

### Retinal: Free-Energy Surface

The free-energy surfaces in this work are computed via umbrella sampling simulations at different bias windows and the weighted histogram analysis method (WHAM).^85,86^ The umbrella sampling simulations are performed using the same MD simulation setup with GROMACS version 2019.6 together with the PLUMED plugin.^87^ The simulations also applies the leapfrog stochastic dynamics integrator with an inverse friction coefficient of 2 ps but a 1 fs time step. The CV is chosen according to the results of the symbolic regression to obtain an approximate expression of the committor function. For each umbrella simulation a harmonic bias with a force constant of 300 kJ/mol/Δ*q* ^2^ is applied, where Δ*q* is the spatial deviation from the respective point along the CV.

The MFEP is computed by the NEB method with 21 beads and spring force of 2 kJ/mol/Å between the *cis* state *A* (θ_1_ = 0, θ_2_ = 0) and the *trans* state *B* (θ_1_ = π, θ_2_ = π) using the 2-dimensional FES.

### TPE Velocity

The dihedral angle velocities in the transition-path ensemble are computed from the atom velocities in Cartesian coordinates ∂**x**/∂*t* stored in the AIMMD-generated trr trajectory files. The partial derivative ∂θ/∂**x** was determined to convert from Cartesian atom velocities to angle velocity by

9 $$\frac{\partial \theta }{\partial t}=\frac{\partial \theta }{\partial x}\frac{\partial x}{\partial t}$$

The projections of the dihedral angle velocities of the TPE along certain angles show the average of the absolute dihedral angle velocities of all frames with retinal-lysine configuration of the respective projected-angle bin.

### pCAA: Training ANI-2x for pCAA

The training data set for the transfer learning of ANI-2x was generated by sampling conformations of the *p*-coumaric acid anion along the dihedral angle θ_1_ from the well-tempered metadynamics simulations described above. The range of θ_1_ from −180° to 180° was divided into 36 bins with a bin width of 10°. From each bin, up to 50 conformations were randomly selected, resulting in a training data set with 1778 conformers with an approximately uniform coverage of the torsional coordinate.

For all selected structures, reference energies and atomic forces were calculated with Gaussian 16 ^88^ using the ωB97X functional and the 6–31G* basis set. The Gaussian route section used for these calculations was #p wB97X/6-31G* Force NoSymm.

For the transfer learning of ANI-2x, the eight neural networks of the ANI-2x ensemble were trained separately. The reference data set was randomly split into training, validation, and test subsets with fractions of 0.8 (1422), 0.1 (178), and 0.1 (178), respectively.

The weights of the element-specific neural networks for H, C, and O were optimized with the AdamW optimizer, while the corresponding bias parameters were optimized with stochastic gradient descent. Both optimizers used an initial learning rate of 1.0 × 10^–4^. Training was performed with a batch size of 128 for a maximum of 5000 epochs and was stopped early when the learning rate decreased below 1.0 × 10^–5^.

The loss function contained contributions from both energies and atomic forces. Forces were obtained as the negative gradient of the predicted energy with respect to the atomic coordinates. The total loss was defined as the sum of the energy mean-squared error and the force mean-squared error weighted by a factor of 0.1. After each epoch, the model was evaluated on the validation set, and the checkpoint with the lowest combined validation RMSE was retained.

### pCAA: AIMMD

The *cis*-*trans* isomerization is investigated for *p*-coumaric acid anion around the C_2_=C_3_ double bond. The state definition function is given by the selected dihedral angle θ_1_ ∈ (−π, π] defined by sp^2^–carbons 1, 2, 3, and 4.

10 $$\mathrm{SDF}\left(\theta_{1}\right)=\{\begin{array}{l} \;A,,\mathrm{if}\;|\theta_{1}|<\frac{\pi }{9},\left(cis\right)\\ B,,\mathrm{if}\;|\theta_{1}|>\pi -\frac{\pi }{9},\left(trans\right)\\ R,,otherwise,\left(reactive\right) \end{array}$$

Here, all two-way shooting MD simulations in AIMMD are not performed by GROMACS but by the OpenMM Python package version 8.2 using a transfer-learned ANI-2x potential model as force field for the *p*-coumaric acid anion.^59,78^ In total, 50,000 two-way shooting simulations are performed with 7479 reactive paths. The two-way shooting simulations are run using the Langevin middle integrator with a 2 fs time step at 300 K with an inverse friction coefficient of 1 ps. The initial atom velocities for the shooting point conformation are randomly samples by OpenMM with respect to the Boltzmann distribution at 300 K. All hydrogen bonds are constrained. The transfer-learned ANI-2x model potential from the TorchAni Python package is linked to OpenMM via OpenMM-ML.^91^

Contrary to retinal, here the input for feedforward neural network is represented by 85 features (19 bonds, 28 angles, and 38 dihedral angles). The ADAM^80^ optimizer is employed for the model training with an initial learning rate equal to *l*_*r*_ = 10^–5^ and an exponential decay scheduler for the learning rate with a decay rate of 0.99 every epoch. The training is performed for 100 epochs with a training batch size of 4096.

Symbolic regression followed the same protocol as for retinal, where we again used the four most significant features.

The free-energy surface for the *p*-coumaric acid anion along two CVs are computed by well-tempered metadynamics simulations using the PLUMED plugin for OpenMM.^87,92–94^ The simulations are performed for 10^6^ time steps at 300 K using the above-mentioned simulation setup with a 1 fs time step. The initial Gaussian bias potential height is set to 1.2 kJ/mol with a width of 0.5° and a bias factor of 8.0 every 10 time steps. Note that the central aim for the metadynamics simulations and its parametrization are to provide conformations of the *p*-coumaric acid anion for reference calculation and not necessarily to yield a well converged free-energy surface.

### pCAA: MFEP, MEP, TS, IRC

The MFEP for the *p*-coumaric acid anion is computed by the NEB method with 21 beads and spring force of 2 kJ/mol/Å between the *cis* state *A* (θ_1_ = 0, θ_2_ = 0) and the *trans* state *B* (θ_1_ = π, θ_2_ = π) using the 2-dimensional FES described in the previous section.

The MEP between the equilibrated *cis* and *trans* configuration of the *p*-coumaric acid anion molecule is predicted by the climbing image nudged elastic band (ci-NEB) method implemented in the atomic simulation environment (ASE) version 3.25.0.^76,77^ The initial path of 17 beads between the *cis* and *trans* configuration is estimated by a scan along the dihedral angle θ_1_ and the spring force between the beads is set to 1 eV/Å. The beads on the path are optimized using the FIRE optimizer implemented in ASE down to a maximum force threshold of below 5 meV/Å. The transfer-learned ANI-2x model potential is used for the energy and force evaluation.

The transition state of the *cis*-*trans* isomerization reaction of the *p*-coumaric acid anion is obtained from the highest potential energy beat of the converged MEP from the ci-NEB calculation. The harmonic analysis of the transition state is performed by the numeric procedure implemented in ASE that yields only one significant imaginary (negative) frequency of −438 cm^–1^.

The intrinsic reaction coordinate is computed by the Sella Python package starting from the transition state with initial forward and reverse displacements along the mode with the imaginary frequency down to a maximum force threshold of below 1 meV/Å.^95^

## Supplementary Material



## Data Availability

AIMMD transition-path sampling data, HIPR and symbolic regression scripts as well as the scripts for data evaluation are available at 10.5281/zenodo.21642855. Data generation and ANI training scripts for pCAA and the scripts for the adapted AIMMD transition-path sampling, HIPR and symbolic regression are provided as well.

## Notes

Derivation of the Bernoulli-trial log-likelihood for committor learning; structural representation and topology of *N*-retinylidene-lysine; committor histogram validation for retinal; projection of the *p*-coumaric acid transition-state ensemble in dihedral-angle space; pairwise correlation analysis of sampled and predicted logit committors for the neural-network and symbolic-regression models (PDF)

## References

[1] Peters, B. Reaction Rate Theory and Rare Events; Elsevier, 2017.

[2] Frenkel, D. ; Smit, B. Understanding Molecular Simulation: From Algorithms to Applications; Elsevier, 2023.

[3] Bolhuis P.
G., Chandler D., Dellago C., Geissler P. L. (2002). Transition path
sampling: Throwing ropes over rough mountain passes, in the dark. Annu. Rev. Phys. Chem..

[4] Peters B. (2016). Reaction coordinates
and mechanistic hypothesis tests. Annu. Rev.
Phys. Chem..

[5] Chen H., Roux B., Chipot C. (2023). Discovering reaction pathways, slow
variables, and committor probabilities with machine learning. J. Chem. Theory Comput..

[6] Gkeka P., Stoltz G., Barati Farimani A., Belkacemi Z., Ceriotti M., Chodera J. D., Dinner A. R., Ferguson A. L., Maillet J.-B., Minoux H. (2020). Machine
learning force
fields and coarse-grained variables in molecular dynamics: application
to materials and biological systems. J. Chem.
Theory Comput..

[7] Beyerle E. R., Mehdi S., Tiwary P. (2022). Quantifying energetic
and entropic
pathways in molecular systems. J. Phys. Chem.
B.

[8] Maragliano L., Fischer A., Vanden-Eijnden E., Ciccotti G. (2006). String method in collective
variables: Minimum free energy paths and isocommittor surfaces. J. Chem. Phys..

[9] de Alba Ortíz, A. P. ; Vreede, J. ; Ensing, B. Biomolecular Simulations: Methods and Protocols; Springer, 2019; pp 255–290.

[10] Leines G. D., Ensing B. (2012). Path finding on high-dimensional
free energy landscapes. Phys. Rev. Lett..

[11] Berezhkovskii A. M., Szabo A. (2019). Committors, first-passage
times, fluxes, Markov states, milestones,
and all that. J. Chem. Phys..

[12] Covino R., Woodside M. T., Hummer G., Szabo A., Cossio P. (2019). Molecular
free energy profiles from force spectroscopy experiments by inversion
of observed committors. J. Chem. Phys..

[13] Kang P., Trizio E., Parrinello M. (2024). Computing the committor with the
committor to study the transition state ensemble. Nat. Comput. Sci..

[14] Hänggi P., Talkner P., Borkovec M. (1990). Reaction-Rate Theory:
Fifty Years
after Kramers. Rev. Mod. Phys..

[15] Metzner P., Schütte C., Vanden-Eijnden E. (2006). Illustration of Transition Path Theory
on a Collection of Simple Examples. J. Chem.
Phys..

[16] Buchete N.-V., Hummer G. (2008). Coarse master equations for peptide folding dynamics. J. Phys. Chem. B.

[17] Schütte C., Noé F., Lu J., Sarich M., Vanden-Eijnden E. (2011). Markov state
models based on milestoning. J. Chem. Phys..

[18] Elber R., Bello-Rivas J. M., Ma P., Cardenas A. E., Fathizadeh A. (2017). Calculating
iso-committor surfaces as optimal reaction coordinates with milestoning. Entropy.

[19] Mitchell A. R., Rotskoff G. M. (2024). Committor guided estimates of molecular transition
rates. J. Chem. Theory Comput..

[20] Ma A., Dinner A. R. (2005). Automatic method
for identifying reaction coordinates
in complex systems. J. Phys. Chem. B.

[21] Arredondo S. C., Tang C., Talmazan R. A., Megías A., Chen C. G., Chipot C. (2026). Learning the committor
without collective
variables. Nat. Comput. Sci..

[22] Kang P., Zhang J., Trizio E., Hou T., Parrinello M. (2026). Committors
without descriptors. J. Chem. Theory Comput..

[23] Khoo Y., Lu J., Ying L. (2019). Solving for
High-Dimensional Committor Functions Using
Artificial Neural Networks. Res. Math. Sci..

[24] Li Q., Lin B., Ren W. (2019). Computing Committor Functions for
the Study of Rare
Events Using Deep Learning. J. Chem. Phys..

[25] Rotskoff, G. M. ; Mitchell, A. R. ; Vanden-Eijnden, E. In Active Importance Sampling for Variational Objectives Dominated by Rare Events, Proceedings of the 2nd Mathematical and Scientific Machine Learning Conference; PMLR, 2022.

[26] Trizio E., Kang P., Parrinello M. (2025). Everything
Everywhere All at Once:
A Probability-Based Enhanced Sampling Approach to Rare Events. Nat. Comput. Sci..

[27] Deng Y., Kang P., Xu X., Li H., Parrinello M. (2026). The Role of
Fluctuations in the Nucleation Process. Proc.
Natl. Acad. Sci. U.S.A..

[28] Strahan J., Guo S. C., Lorpaiboon C., Dinner A. R., Weare J. (2023). Inexact Iterative
Numerical Linear Algebra for Neural Network-Based Spectral Estimation
and Rare-Event Prediction. J. Chem. Phys..

[29] Peters B., Trout B. L. (2006). Obtaining reaction coordinates by likelihood maximization. J. Chem. Phys..

[30] Hummer G. (2004). From transition
paths to transition states and rate coefficients. J. Chem. Phys..

[31] Jung H., Covino R., Arjun A., Leitold C., Dellago C., Bolhuis P. G., Hummer G. (2023). Machine-guided path sampling to discover
mechanisms of molecular self-organization. Nat.
Comput. Sci..

[32] Lazzeri G., Jung H., Bolhuis P. G., Covino R. (2023). Molecular Free Energies,
Rates, and Mechanisms from Data-Efficient Path Sampling Simulations. J. Chem. Theory Comput..

[33] Jackel E., Lazzeri G., Covino R. (2025). Free energy, rates,
and mechanism
of transmembrane dimerization in lipid bilayers from dynamically unbiased
molecular dynamics simulations. J. Phys. Chem.
B.

[34] Post M., Hummer G. (2026). AI-guided transition path sampling of lipid flip-flop
and membrane nanoporation. Nat. Commun..

[35] Horvath F., Jung H., Grabmayr H., Fahrner M., Romanin C., Hummer G. (2025). STIM1 transmembrane helix dimerization captured by
AI-guided transition path sampling. Proc. Natl.
Acad. Sci. U.S.A..

[36] Brünig, F. N. ; Dalton, B. A. ; Daldrop, J. O. ; Netz, R. R. Non-Markovianity increases transition path probability, arXiv:2501.04512. arXiv.org e-Print archive, 2025 https://arxiv.org/abs/2501.04512.

[37] Roux B. (2021). String method
with swarms-of-trajectories, mean drifts, lag time, and committor. J. Phys. Chem. A.

[38] E W., Vanden-Eijnden E. (2010). Transition-path theory and path-finding
algorithms
for the study of rare events. Annu. Rev. Phys.
Chem..

[39] France-Lanord A., Vroylandt H., Salanne M., Rotenberg B., Saitta A. M., Pietrucci F. (2024). Data-driven path collective variables. J. Chem. Theory Comput..

[40] Mullen R. G., Shea J.-E., Peters B. (2014). Transmission Coefficients,
Committors,
and Solvent Coordinates in Ion-Pair Dissociation. J. Chem. Theory Comput..

[41] Polanyi J. C. (1987). Some concepts
in reaction dynamics. Science.

[42] Miller W. H. (1993). Beyond
transition-state theory: a rigorous quantum theory of chemical reaction
rates. Acc. Chem. Res..

[43] Herschbach D. R. (1987). Molecular
dynamics of elementary chemical reactions (Nobel Lecture). Angew. Chem., Int. Ed..

[44] Breebaart, R. S. ; Lazzeri, G. ; Covino, R. ; Bolhuis, P. G. Understanding Reaction Mechanisms from Start to Finish, arXiv:2507.04052. arXiv.org e-Print archive, 2025 https://arxiv.org/abs/2507.04052.

[45] Makke N., Chawla S. (2024). Interpretable scientific
discovery with symbolic regression:
a review. Artif. Intell. Rev..

[46] Ghysbrecht S., Donati L., Keller B. G. (2025). Accuracy of reaction
coordinate based
rate theories for modelling chemical reactions: insights from the
thermal isomerization in retinal. J. Comput.
Chem..

[47] Ghysbrecht S., Keller B. G. (2024). Thermal isomerization rates in retinal analogues using
Ab-Initio molecular dynamics. J. Comput. Chem..

[48] Schlichting I., Berendzen J. (1997). Out of the
blue: the photocycle of the photoactive
yellow protein. Structure.

[49] Aristoff D., Johnson M., Simpson G., Webber R. J. (2024). A Weight-Averaged,
Adaptive Stratification Scheme for Estimating Rare-Event Rates and
Committors. J. Chem. Phys..

[50] Dellago C., Bolhuis P. G., Chandler D. (1998). Efficient transition path sampling:
Application to Lennard-Jones cluster rearrangements. J. Chem. Phys..

[51] Falkner S., Coretti A., Peters B., Bolhuis P. G., Dellago C. (2025). Revisiting
shooting point Monte Carlo methods for transition path sampling. J. Chem. Phys..

[52] Lazzeri, G. ; Bolhuis, P. G. ; Covino, R. Optimal Rejection-Free Path Sampling, arXiv:2503.21037. arXiv.org e-Print archive, 2025 https://arxiv.org/abs/2503.21037.

[53] Van
Erp T. S., Moroni D., Bolhuis P. G. (2003). A novel path sampling
method for the calculation of rate constants. J. Chem. Phys..

[54] Banushkina P. V., Krivov S. V. (2016). Optimal reaction coordinates. WIREs Comput. Mol. Sci..

[55] Berezhkovskii A. M., Szabo A. (2013). Diffusion along the Splitting/Commitment Probability Reaction Coordinate. J. Phys. Chem. B.

[56] Bolhuis P. G., Dellago C., Chandler D. (2000). Reaction Coordinates of Biomolecular
Isomerization. Proc. Natl. Acad. Sci. U.S.A..

[57] Kemp S. J., Zaradic P., Hansen F. (2007). An approach
for determining relative
input parameter importance and significance in artificial neural networks. Ecol. Modell..

[58] Malmerberg E., Omran Z., Hub J. S., Li X., Katona G., Westenhoff S., Johansson L. C., Andersson M., Cammarata M., Wulff M. (2011). Time-resolved
WAXS reveals
accelerated conformational changes in iodoretinal-substituted proteorhodopsin. Biophys. J..

[59] Devereux C., Smith J. S., Huddleston K. K., Barros K., Zubatyuk R., Isayev O., Roitberg A. E. (2020). Extending
the applicability of the
ANI deep learning molecular potential to sulfur and halogens. J. Chem. Theory Comput..

[60] Fukui K. (1981). The path of
chemical reactions—the IRC approach. Acc. Chem. Res..

[61] Gonzalez C., Schlegel H. B. (1990). Reaction path following
in mass-weighted internal coordinates. J. Phys.
Chem. A.

[62] Evans M. G., Polanyi M. (1935). Some applications of the transition state method to
the calculation of reaction velocities, especially in solution. Trans. Faraday Soc..

[63] Eyring H. (1935). The activated
complex in chemical reactions. J. Chem. Phys..

[64] Hartke B., Manz J. (1988). Do chemical reactions
react along the reaction path?. J. Am. Chem.
Soc..

[65] a
Beccara S., Faccioli P., Sega M., Pederiva F., Garberoglio G., Orland H. (2011). Dominant folding pathways of a peptide
chain from ab initio quantum-mechanical simulations. J. Chem. Phys..

[66] Lemke O., Keller B. G. (2016). Density-based cluster
algorithms for the identification
of core sets. J. Chem. Phys..

[67] Rogal J., Bolhuis P. G. (2008). Multiple state transition
path sampling. J. Chem. Phys..

[68] Lindorff-Larsen K., Piana S., Palmo K., Maragakis P., Klepeis J. L., Dror R. O., Shaw D. E. (2010). Improved side-chain
torsion potentials for the Amber ff99SB protein force field. Proteins: Struct., Funct., Bioinf..

[70] Hub, J. S. GROMACS topology for retinal 2011 https://biophys.uni-saarland.de/software-auxiliary/retinal/. (last visited 03.02.2026).

[71] Hayashi S., Tajkhorshid E., Schulten K. (2002). Structural Changes during the Formation
of Early Intermediates in the Bacteriorhodopsin Photocycle. Biophys. J..

[72] Volkov O., Kovalev K., Polovinkin V., Borshchevskiy V., Bamann C., Astashkin R., Marin E., Popov A., Balandin T., Willbold D., Büldt G., Bamberg E., Gordeliy V. (2017). Structural insights
into ion conduction
by channelrhodopsin 2. Science.

[73] Van
Der Spoel D., Lindahl E., Hess B., Groenhof G., Mark A. E., Berendsen H. J. C. (2005). GROMACS: Fast, flexible, and free. J. Comput. Chem..

[74] Abraham M. J., Murtola T., Schulz R., Páll S., Smith J. C., Hess B., Lindahl E. (2015). GROMACS: High performance
molecular simulations through multi-level parallelism from laptops
to supercomputers. SoftwareX.

[75] Hess B., Bekker H., Berendsen H. J. C., Fraaije J. G. E. M. (1997). LINCS: A linear
constraint solver for molecular simulations. J. Comput. Chem..

[76] Larsen A. H., Mortensen J. J., Blomqvist J., Castelli I. E., Christensen R., Dułak M., Friis J., Groves M. N., Hammer B., Hargus C. (2017). The atomic simulation environment—a Python library
for working with atoms. J. Phys.: Condens. Matter.

[77] Henkelman G., Uberuaga B. P., Jónsson H. (2000). A climbing
image nudged elastic band
method for finding saddle points and minimum energy paths. J. Chem. Phys..

[78] Eastman P., Galvelis R., Peláez R. P. (2024). OpenMM 8: Molecular
Dynamics Simulation with Machine Learning Potentials. J. Phys. Chem. B.

[79] O’Connor M. B., Bennie S. J., Deeks H. M., Jamieson-Binnie A., Jones A. J., Shannon R. J., Walters R., Mitchell T. J., Mulholland A. J., Glowacki D. R. (2019). Interactive molecular dynamics in
virtual reality from quantum chemistry to drug binding: An open-source
multi-person framework. J. Chem. Phys..

[80] Kingma, D. P. ; Ba, J. Adam: A Method for Stochastic Optimization, arXiv:1412.6980. arXiv.org e-Print archive, 2017 https://arxiv.org/abs/1412.6980.

[81] Falkner S., Coretti A., Romano S., Geissler P. L., Dellago C. (2023). Conditioning
Boltzmann generators for rare event sampling. Mach. Learn.: Sci. Technol..

[82] Best R. B., Hummer G. (2005). Reaction coordinates and rates from
transition paths. Proc. Natl. Acad. Sci. U.S.A..

[83] Paszke, A. ; Gross, S. ; Massa, F. PyTorch: An Imperative Style, High-Performance Deep Learning Library, arXiv:1912.01703. arXiv.org e-Print archive, 2019 https://arxiv.org/abs/1912.01703.

[84] Cranmer, M. Interpretable Machine Learning for Science with PySR and SymbolicRegression.jl, arXiv:2305.01582. arXiv.org e-Print archive, 2023 https://arxiv.org/abs/2305.01582.

[85] Kumar S., Rosenberg J. M., Bouzida D., Swendsen R. H., Kollman P. A. (1992). The weighted
histogram analysis method for free-energy calculations on biomolecules.
I. The method. J. Comput. Chem..

[86] Souaille M., Roux B. (2001). Extension to the weighted
histogram analysis method: combining umbrella
sampling with free energy calculations. Comput.
Phys. Commun..

[87] Tribello G. A., Bonomi M., Branduardi D., Camilloni C., Bussi G. (2014). PLUMED 2: New feathers for an old
bird. Comput.
Phys. Commun..

[88] Frisch, M. J. ; Trucks, G. W. ; Schlegel, H. B. In Gaussian 16, Revision C.01; Gaussian Inc.: Wallingford CT, 2016.

[91] Gao X., Ramezanghorbani F., Isayev O., Smith J. S., Roitberg A. E. (2020). TorchANI:
A Free and Open Source PyTorch-Based Deep Learning Implementation
of the ANI Neural Network Potentials. J. Chem.
Inf. Model..

[92] Barducci A., Bussi G., Parrinello M. (2008). Well-Tempered Metadynamics: A Smoothly
Converging and Tunable Free-Energy Method. Phys.
Rev. Lett..

[93] Zhang S., Makoś M. Z., Jadrich R. B., Kraka E., Barros K., Nebgen B. T., Tretiak S., Isayev O., Lubbers N., Messerly R. A., Smith J. S. (2024). Exploring the frontiers of condensed-phase
chemistry with a general reactive machine learning potential. Nat. Chem..

[94] Bonomi M., Branduardi D., Bussi G., Camilloni C., Provasi D., Raiteri P., Donadio D., Marinelli F., Pietrucci F., Broglia R. A., Parrinello M. (2009). PLUMED: A
portable plugin for free-energy calculations with molecular dynamics. Comput. Phys. Commun..

[95] Hermes E. D., Sargsyan K., Najm H. N., Zádor J. (2022). Sella, an
Open-Source Automation-Friendly Molecular Saddle Point Optimizer. J. Chem. Theory Comput..

