# Supplementary material for: A Machine-Learned Symbolic Committor for a Chemical Reaction: Isomerization in Retinal and p‑Coumaric Acid
Source: J Chem Theory Comput. 2026 Oct 30;22(21):11233–50. doi: 10.1021/acs.jctc.6c01496 (PMC13757763; doi:10.1021/acs.jctc.6c01496)
Supplement: Supplementary file 1 [file ct6c01496_si_001.pdf]

Supporting Information: A Machine-Learned Symbolic Committor for a Chemical Reaction: Isomerization in Retinal and p-Coumaric Acid

Kai Töpfer,[†,‡] Gianmarco Lazzeri,[¶,§,‡] Vittoria Ossanna,[‖,§] Christopher J. I. Bläß,[†] Florian Renner,[†] Gianluca Lattanzi,[⊥] Roberto Covino,[¶,§] and Bettina G. Keller[*,†]

†*Department of Biology, Chemistry and Pharmacy, Freie Universität Berlin, 14195 Berlin, Germany.*

‡*These authors contributed equally to this work.*

¶*Institute of Computer Science, Goethe University Frankfurt, Frankfurt am Main, Germany.*

§*Frankfurt Institute for Advanced Studies, Frankfurt am Main, Germany.*

‖*Department of Cellular, Computational and Integrative Biology, University of Trento, Trento, Italy.*

⊥*Department of Physics, University of Trento, Trento, Italy.*

E-mail: bettina.keller@fu-berlin.de,covino@fias.uni-frankfurt.de

Log-likelihood of a Bernoulli-trial

For k independent Bernoulli trials, where each trial has its own probability $p_B(\mathbf{x}_i|\mathbf{w})$, the negative log likelihood is

$$
\begin{aligned}
-\ln P(S|\mathbf{w}) &= -\ln \prod_{i=1}^{k} p_B(\mathbf{x}_i|\mathbf{w})^{\delta_i} \cdot (1 - p_B(\mathbf{x}_i|\mathbf{w}))^{1-\delta_i} \\
&= \sum_{i=1}^{k} \delta_i \left(-\ln p_B(\mathbf{x}_i|\mathbf{w})\right) + (1-\delta_i)\left(-\ln(1 - p_B(\mathbf{x}_i|\mathbf{w}))\right) \\
&= \sum_{i=1}^{k} \delta_i \ln(1 + e^{-q(\mathbf{x}_i|\mathbf{w})}) + (1-\delta_i) \ln(1 + e^{+q(\mathbf{x}_i|\mathbf{w})}) \\
&= \sum_{i=1}^{k} \delta_i \ln(1 + e^{s_i q(\mathbf{x}_i|\mathbf{w})}) + (1-\delta_i) \ln(1 + e^{s_i q(\mathbf{x}_i|\mathbf{w})}) \\
&= \sum_{i=1}^{k} \ln(1 + e^{s_i q(\mathbf{x}_i|\mathbf{w})}) \qquad (1)
\end{aligned}
$$

where δ_i is a binary outcome variable with $\delta_i = 1$ if the trajectory reaches B before A, and $\delta_i = 0$ otherwise. The signed outcome variable is $s_i = -1$ if trajectory i reaches B before A, and $s_i = +1$ if it reaches A before B. The two outcome variables are related by $s_i = 1 - 2\delta_i$. In Eq. 1 we used the identities in Eq. 2

$$
\begin{aligned}
-\ln p_B &= -\ln \frac{1}{1+e^{-q}} = \ln(1 + e^{-q}) \\
-\ln(1 - p_B) &= -\ln\left(1 - \frac{1}{1+e^{-q}}\right) = -\ln\left(\frac{e^{-q}}{1+e^{-q}}\right) = \ln\left(\frac{1+e^{-q}}{e^{-q}}\right) = \ln(1 + e^{+q})\,. \qquad (2)
\end{aligned}
$$

Here, we abbreviated $p_B(\mathbf{x}) = p_B$ and $q_B(\mathbf{x}) = q$.

Figures

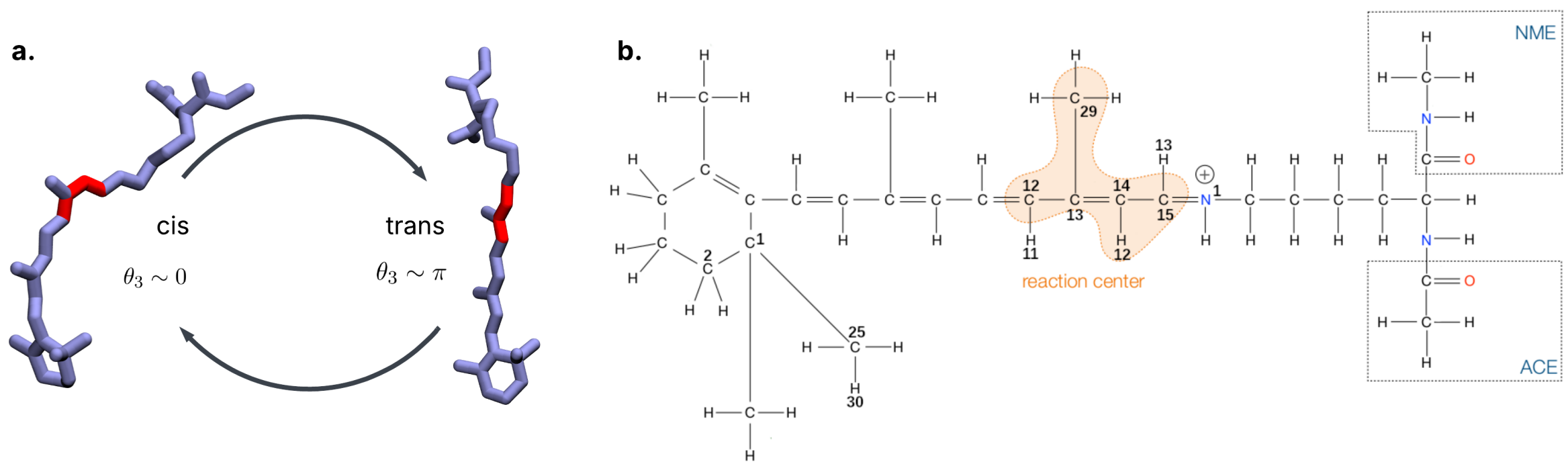

Figure S1: **(a.)** *cis*-*trans* isomerization of N-retinylidene-lysine **(b.)** Topology of the molecule with reaction center highlighted.

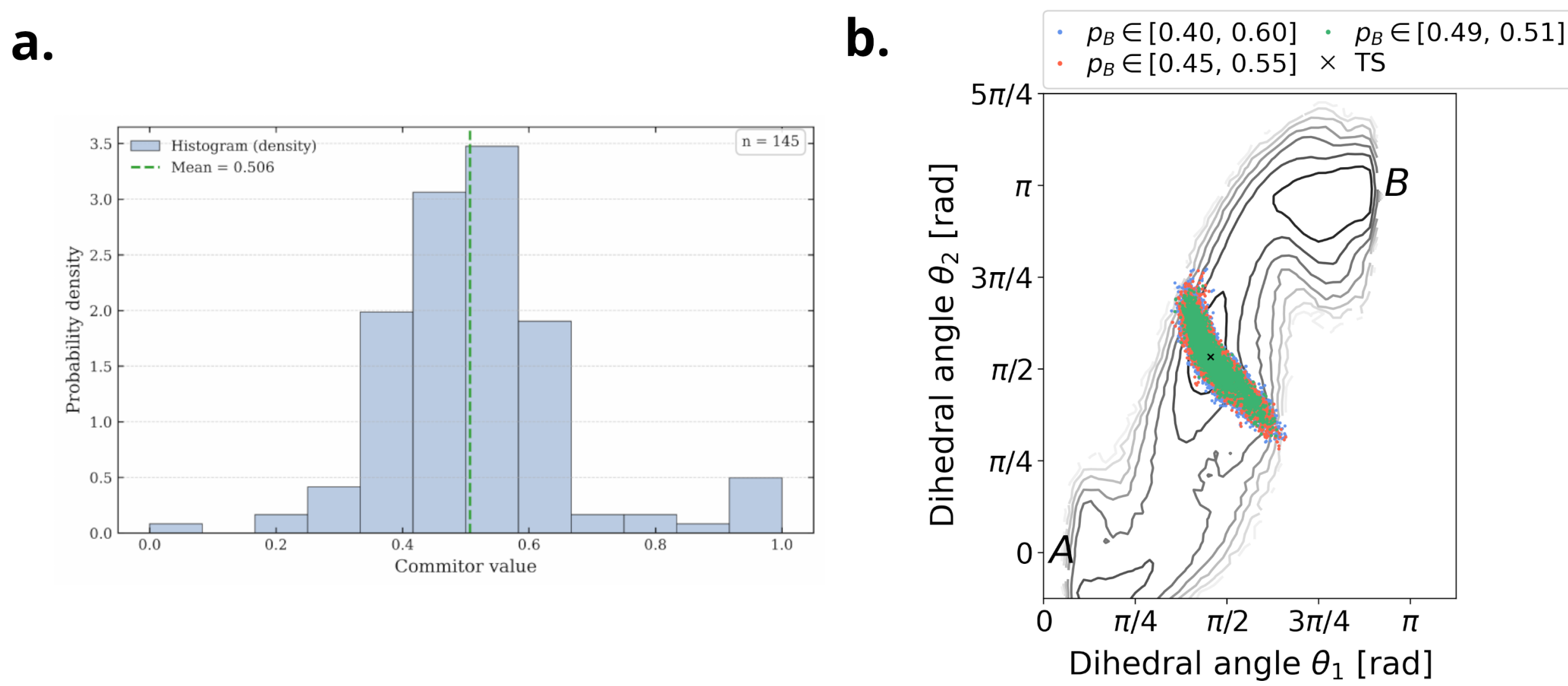

Figure S2: **(a.)**: Committor histogram test for retinal linked to a lysine residue . A total of 145 retinal conformations with predicted committor values near 0.5 were sampled. Their corresponding estimated committor values were obtained from repeated two-way shooting simulations following the same protocol as for the validation of the neural-network committor (See Methods → NN embedding of the committor). This figure shows a histogram of the resulting 145 estimated committor values, which closely follows a normal distribution, with a mean committor value of 0.506. **(b.)**: Projection of p-coumaric acid configuration ensemble near the transition state into (θ_1, θ_2)-dihedral angle space by different colored point clouds. The transition-path ensemble projection as shown in Fig. 7.a. is shown by contour lines for visual orientation.

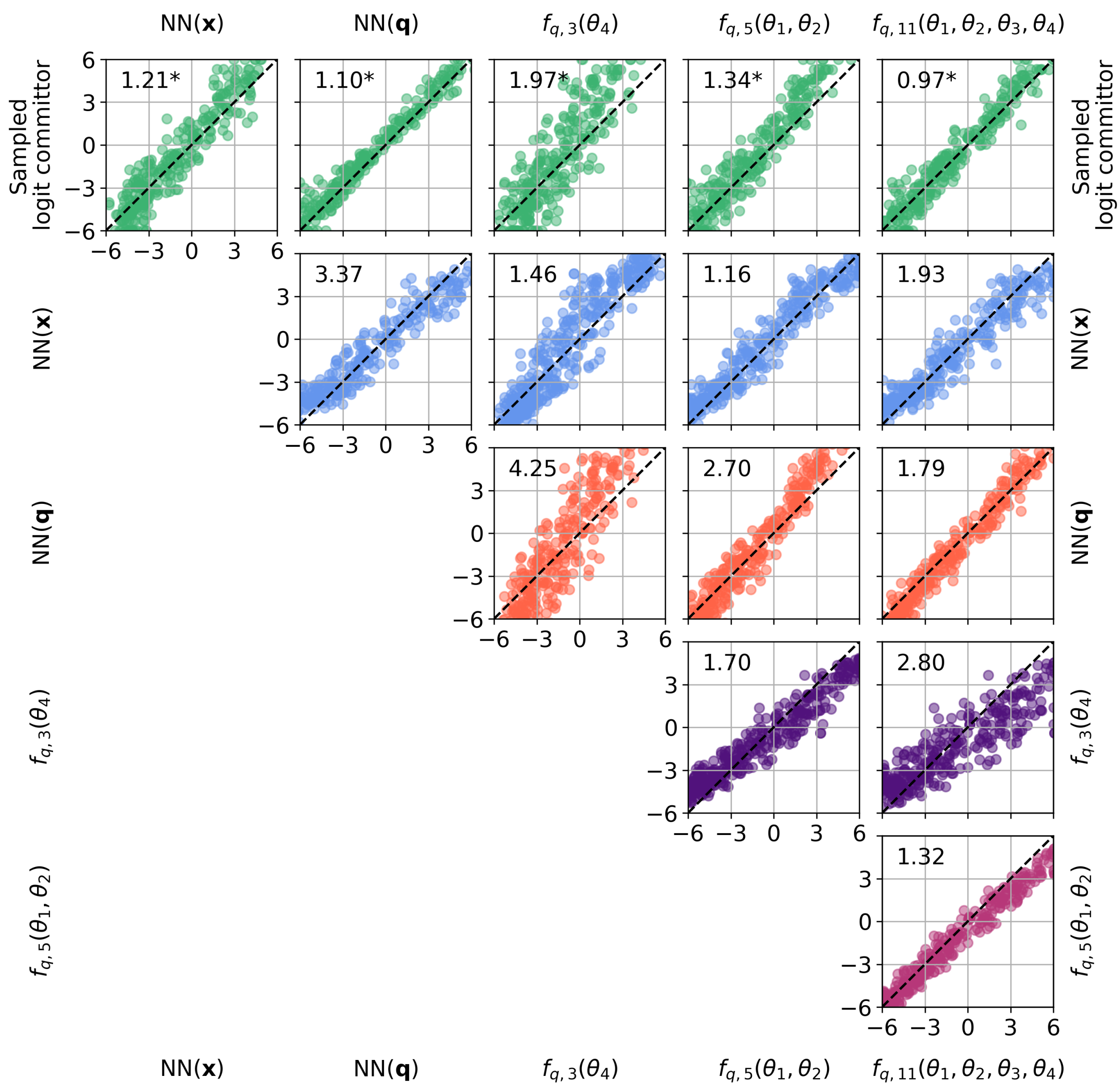

Figure S3: Pairwise correlations between the sampled logit committor values and our 5 logit committor models: NN($\mathbf{x}$): NN logit committor model based on atom pair distances, NN($\mathbf{q}$): NN logit committor model based on internal coordinates, and the SR solutions $f_{q,3}(\theta_4)$, $f_{q,5}(\theta_1, \theta_2)$, $f_{q,11}(\theta_1, \theta_2, \theta_3, \theta_4)$ for the logit committor prediction. The top-left value denotes the RMSE between the two logit committors. Values marked * do not include sampled committor values of zero or one (175 out of 438), for which the logit function is not defined.
